# Comprehensive assessment of computational methods for cancer immunoediting

**DOI:** 10.1016/j.crmeth.2025.101006

**Published:** 2025-03-24

**Authors:** Shengyuan He, Shangqin Sun, Kun Liu, Bo Pang, Yun Xiao

**Affiliations:** 1 College of Bioinformatics Science and Technology, Harbin Medical University, Harbin, Heilongjiang 150081, China

**Keywords:** immunoediting, computational methods, immune selection

## Abstract

Cancer immunoediting reflects the role of the immune system in eliminating tumor cells and shaping tumor immunogenicity, which leaves marks in the genome. In this study, we systematically evaluate four methods for quantifying immunoediting. In colorectal cancer samples from The Cancer Genome Atlas, we found that these methods identified 78.41%, 46.17%, 36.61%, and 4.92% of immunoedited samples, respectively, covering 92.90% of all colorectal cancer samples. Comparison of 10 patient-derived xenografts (PDXs) with their original tumors showed that different methods identified reduced immune selection in PDXs ranging from 44.44% to 60.0%. The proportion of such PDX-tumor pairs increases to 77.78% when considering the union of results from multiple methods, indicating the complementarity of these methods. We find that observed-to-expected ratios highly rely on neoantigen selection criteria and reference datasets. In contrast, HLA-binding mutation ratio, immune dN/dS, and enrichment score of cancer cell fraction were less affected by these factors. Our findings suggest integration of multiple methods may benefit future immunoediting analyses.

## Highlights

• We assess four quantitative methods for cancer immunoediting
• These methods show complementarity in identifying immunoedited samples
• Immunoediting scores derived from these methods are influenced by different factors

## Motivation

Accurate quantification of tumor immunoediting signatures is anticipated to advance the development of immunotherapy and precision medicine. Several methods, including immunogenic rate-based observed-to-expected ratio (OE-ratio), HLA-binding mutation ratio (HBMR), immune dN/dS, and enrichment score of cancer cell fraction (ES-CCF), have been developed to quantify immunoediting, but a systematic assessment of these methods remains lacking.

## Graphical abstract

**Figure:**
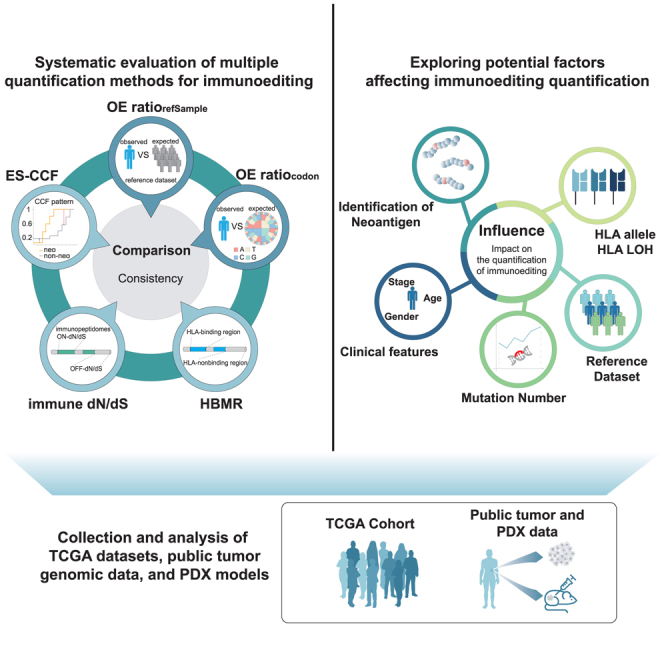

## Teaser

He et al. systematically evaluate four methods for quantifying immunoediting using genomic data from the TCGA cohort, additional cancer samples, and patient-derived xenograft models. Several factors can influence the quantification of immunoediting by these methods. This study will help researchers better understand and apply these computational methods for immunoediting.

## Introduction

Cancer development involves a complex interaction between tumor cells and the immune system, a process known as “cancer immunoediting.” It is characterized by three phases: elimination, equilibrium, and escape, during which the immune system not only inhibits tumor growth but also shapes the immunogenicity of tumor cells.^1^^,^^2^^,^^3^ While the immune system eliminates continuously arising immunogenic tumor cells, persistent immune selection promotes the growth of low-immunogenic tumors, leading to a more malignant phenotype.^4^^,^^5^ Characterizing immunoediting helps to elucidate tumor evolution in the context of tumor immune interaction, providing new insights to improve therapeutic approaches like immunotherapy.^6^^,^^7^^,^^8^

In 2015, Rooney and colleagues were the first to measure the immunoediting scores in The Cancer Genome Atlas (TCGA) samples by comparing the observed neoantigens with non-synonymous mutations ratio with their expected values and found pronounced neoepitope depletion in colorectal and renal clear cell carcinomas.^9^ This method was later applied to ovarian cancer,^10^ hepatocellular carcinoma,^11^ and other cancers,^8^^,^^12^^,^^13^ and further modified by Angelova et al., who utilized the probability of non-synonymous codons to calculate the expected ratio of neoantigens to non-synonymous mutations, revealing an association between immunoediting and recurrence risk.^14^ Van den Eynden et al. developed the HLA-binding mutation ratio (HBMR) method by annotating HLA-binding and non-binding regions of the genome and comparing non-synonymous to synonymous mutations. They found untreated tumors exhibited weak immunoediting scores.^15^ Immune dN/dS is also a genomic region-based method that uses patient-specific HLA genotypes to annotate the immunopeptidome, which generates peptides natively exposed to the immune system and measures immunoediting by calculating dN/dS on the immunopeptidome.^16^ Additionally, a recent study quantified immunoediting by comparing distribution patterns of the cancer cell fraction (CCF) of antigenic mutations (neoantigens) and non-antigenic mutations and explored its potential application in immunotherapy.^17^ Despite the variety of methods to quantify immunoediting, a systematic assessment of them is still lacking. A thorough understanding of the factors influencing them could help guide researchers in selecting appropriate methods.

Here, we compared four computational methods for quantifying cancer immunoediting^9^^,^^14^^,^^15^^,^^16^^,^^17^ using 23 TCGA epithelial cancers^18^ and xenograft models generated in immunodeficient mice.^19^ Furthermore, the potential impact of neoantigen selection strategies, mutation counts, HLA alleles, clinical features, and reference sets on these methods was also analyzed. Taken together, our study revealed various properties of these computational methods and offered some guidance for their rational application.

## Result

### Overview of the quantitative methods for immunoediting

**Figure 1:**
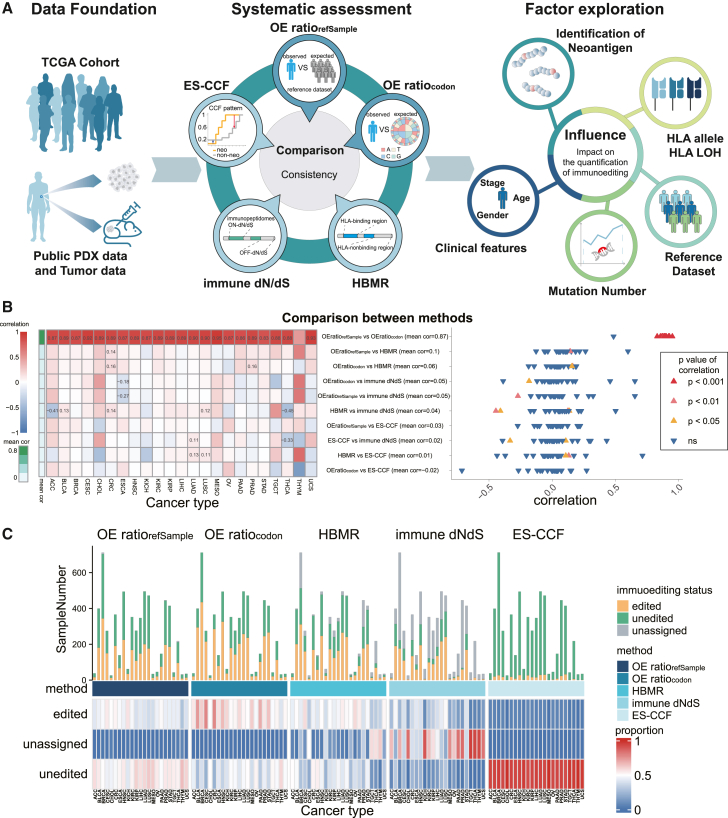
Schematic of the study and assessment based on TCGA multiple epithelial cancers (A) Study overview. First, genome data from TCGA, publicly available PDX models, and an additional cohort were collected. Second, immunoediting scores calculated by four types of methods (including five specific methods) are compared to assess the consistency of these methods in measuring immunoediting scores and the ability to detect immunoediting events in tumors. Finally, the potential impact of neoantigen selection strategies, mutation counts, HLA alleles, clinical features, and reference datasets on the performance of these methods is assessed. (B) Left: Spearman correlation between immunoediting scores derived from different immunoediting quantification methods across various cancer types. Each column represents a cancer type, and each row represents the comparison between two methods. Correlation coefficients with *p* < 0.05 are shown in each cell. Right: The distribution of correlation between methods, the shape of the point indicates the significance of correlation (triangle for significant correlations and inverted triangle for non-significant ones). (C) Top: The number of samples classified by each method as edited, unedited, or unassigned across multiple cancer types. Bottom: The proportion of each classification status by the four methods across each cancer type. Abbreviations: TCGA, The Cancer Genome Atlas; PDX, patient-derived xenograft; HLA, human leukocyte antigen. See also Table S1.

Our study evaluated four methods for quantifying cancer immunoediting (Table 1; Figure 1A). The central assumption of these methods is that immune-mediated negative selection eliminates neoantigen-carrying tumor cells, resulting in fewer neoantigens or non-synonymous mutations than expected. The immunogenic rate–based observed-to-expected ratios (OE-ratios, including ${OE\;ratio}_{refSample}$ and ${OE\;ratio}_{codon}$) focus on changes in the neoantigens to non-synonymous mutations ratio. In particular, ${OE\;ratio}_{refSample}$ relies entirely on reference datasets to calculate the expected number of neoantigens and non-synonymous mutations, while ${OE\;ratio}_{codon}$ estimates the number of non-synonymous mutations using the probability that the mutations' trinucleotide context will produce non-synonymous codons.^9^^,^^14^ If the observed value was a lower than expected value (i.e., OE-ratio <1), immunoediting of the sample is deemed to have occurred. HLA-binding mutation ratio (HBMR) compares non-synonymous to synonymous mutation ratios in HLA-binding (genomic regions that can produce HLA-binding peptides) vs. non-binding regions (genomic regions that cannot produce HLA-binding peptides) to measure immunoediting.^15^ Another genomic region-based method, immune dN/dS, leverages patient-specific HLA genotypes to annotate the immunopeptidome capable of generating peptides natively exposed to the immune system and quantifies immunoediting by assessing variations in dN/dS between the immunopeptidome and non-immunopeptidome.^16^ When immune dN/dS is below 1, the sample is considered to have undergone immunoediting (i.e., the dN/dS inside the immunopeptidome is lower than that outside the immunopeptidome). Enrichment score–CCF (ES-CCF) proposes that the elimination of neoantigen-bearing tumor cells leads to the down-regulation of the CCF of neoantigens and the CCF of non-antigenic mutations is used as a reference to quantify immunoediting.^17^ Reproduced immunoediting scores based on these methods showed strong correlation with the original results (Figure S1), which demonstrated that we could use these methods to reproduce previous results properly.

**Table 1:** Summary of computational methods of cancer immunoediting included in this study

Method		Categorization	Characteristic	Reference
Immunogenic rate–based observed-to-expected ratio	complete dependence on reference datasets (${OE\;ratio}_{refSample}$)	comparing observed and expected neoantigen and non-synonymous mutations	1. using a reference dataset to count expected neoantigens and non-synonymous mutations 2. focus on the entire genome 3. consider 192 mutation contexts	Rooney et al.,^9^ Jimenez-Sanchez et al.^10^ and Zhang et al.^20^
	partial dependence on reference datasets (${OE\;ratio}_{codon}$)		1. using a reference dataset to count expected neoantigens 2. counting non-synonymous mutations by theoretical probabilities 3. focus on the entire genome 4. consider 192 mutation contexts	Angelova et al.^14^
Genomic region-based methods	HLA-binding mutation ratio (HBMR)	comparing non-synonymous and synonymous mutations in HLA-binding vs. non-binding regions	1. no reliance on reference datasets 2. focus on certain regions of the genome 3. consider 96 mutation contexts	Van den Eynden et al.^15^
	immune dN/dS	comparing the dN/dS on and off immunopeptidome	1. no reliance on reference datasets 2. focus on certain regions of the genome 3. consider 192 mutation contexts	Zapata et al.^16^
Enrichment score of CCF (ES-CCF)		comparing distribution patterns of CCF between antigenic and non-antigenic mutations	1. using CCF to gauge immune selection pressure 2. focus on the entire genome 3. no consideration of mutation contexts	Wu et al.^17^

### Systematic comparison of consistency among methods through TCGA epithelial cancers

To assess the consistency of these methods, we applied them to measure the immunoediting score of 5,869 in epithelial cancer samples (Table S1) and compared the correlation of the scores derived from each method. A strong positive correlation existed between methods based on observed-to-expected neoantigen ratio across most cancer types, with a mean Spearman correlation coefficient of 0.87, while there was a negligible mean Spearman correlation of 0.01 between HBMR and ES-CCF (Figure 1B). An exception was Thymoma (THYM), where HBMR and ES-CCF showed a higher correlation (Spearman correlation coefficient 0.71, *p* < 0.05) compared with the correlation between the two OE-ratios (Spearman correlation coefficient 0.49, *p* < 0.05), and the correlation between OE-ratios and immune dN/dS was increased, with Spearman correlation of 0.65 for ${OE\;ratio}_{refSample}$ and 0.65 for ${OE\;ratio}_{codon}$ (*p* < 0.05). In addition, OE-ratios showed slight but significant correlation with HBMR, immune dN/dS, and ES-CCF only in adrenocortical carcinoma (ACC), bladder urothelial carcinoma (BLCA), colorectal cancer (CRC), esophageal carcinoma (ESCA), lung adenocarcinoma (LUAD), lung squamous cell carcinoma (LUSC), prostate adenocarcinoma (PRAD), and thyroid carcinoma (THCA, Figure 1B).

We then identified immunoedited samples based on immunoediting scores measured by these methods. OE-ratios detected a higher proportion of samples that experienced immunoediting across various cancers than HBMR, immune dN/dS, or ES-CCF (Figure 1C). Genomic region-based methods, particularly immune dN/dS, may not effectively quantify immunoediting in samples with limited mutations, which may lack available mutations in binding (immunopeptidome) or non-binding regions (non-immunopeptidome). Compared with other methods, ES-CCF identified a substantial proportion of samples as "unedited" (Figure 1C).

**Figure 2:**
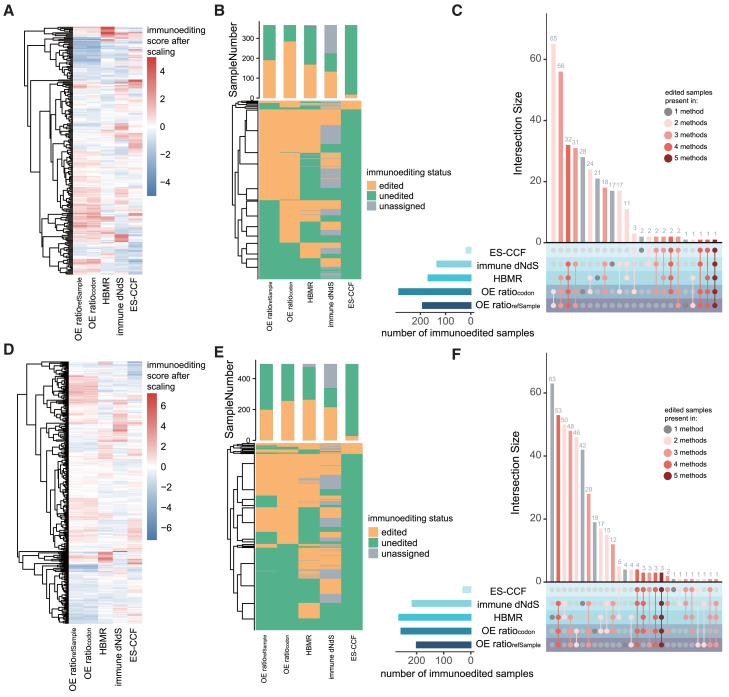
In-depth assessments of various methods based on colorectal cancer and lung adenocarcinoma (A and D) Heatmap shows the similarity in immunoediting scores calculated by different methods across TCGA CRC samples (A) and LUAD samples (D). (B and E) Bar chart and heatmap show the total number of samples classified by each method as edited, unedited, or unassigned in CRC (B) and LUAD (E). (C and F) UpSet plot shows the intersection of immunoedited samples identified by different methods in CRC (C) and LUAD (F). The left bar plot shows the total number of immunoedited samples per method. The top bar plot shows the shared samples among methods, with color indicating the number of methods involved. See also Figures S2 and S3.

In order to make detailed comparisons, we subsequently conducted a further analysis in colorectal cancer from TCGA (CRC, *n* = 366). Compared with the average correlation observed across multiple cancer types, the correlation between two OE-ratios remained strong (Spearman correlation coefficient 0.89, *p* < 0.05, Figure 2A; Figure S2A), while their correlation with HBMR increased (Spearman correlation coefficient 0.14 for ${OE\;ratio}_{refSample}$ and 0.16 for ${OE\;ratio}_{codon}$, respectively, *p* < 0.05, Figures 2A and S2A). Slight but significant correlations were observed between HBMR and immune dN/dS (Spearman correlation coefficient 0.14, *p* < 0.05, Figure S2A). Despite the high degree of concordance of between two OE-ratios, ${OE\;ratio}_{codon}$ was usually lower than ${OE\;ratio}_{refSample}$ (*p* < 0.001, two-sided Wilcoxon rank-sum tests, Figures S2B and S2C). ${OE\;ratio}_{codon}$ also showed a higher expected neoantigen-to-non-synonymous mutation ratio (Figure S2D), which might lead to stronger immunoediting measured under identical observations, making it more likely to classify the sample as immunoedited (Figure 2B). Overall, these methods identified 78.41% (287 of 366, OE-ratios), 46.17% (169 of 366, HBMR), 36.61% (134 of 366, immune dN/dS), and 4.92% (18 of 366, ES-CCF) of immunoedited samples respectively, covering 92.90% (340 of 366) of samples (Figure 2C). While only 0.88% (3/340) of the samples were uniformly identified as edited across all four methods, a larger proportion of the immunoedited samples (15.59% [53/340]) was uniformly identified by OE-ratios, HBMR, and immune dN/dS (Figure 2C).

Except for ES-CCF and immune dN/dS, the other methods did not consider the significance of immunoediting scores. We next assessed the significance of OE-ratios and HBMR by randomly permuting all mutations or HLA-binding and non-binding sites (*n* = 1,000) (see STAR Methods). With the exception of immune dN/dS (53.8%), only a small fraction of TCGA CRC samples exhibited significant immunoediting scores for the remaining methods (range 4%–11%, Figure S2E). Besides, without considering significance, the ES-CCF increases the number of samples identified as edited (Figure S2F).

Overall, these results demonstrate the substantial overlap of immunoedited samples identified by OE-ratios, HBMR, and immune dN/dS, while the stringent significance threshold of ES-CCF likely contributes to its tendency to classify samples as unedited.

### Validation across diverse cancer types and additional datasets

Previous studies have shown that cancers, as complex diseases, exhibit diverse immune profiles across and within cancer types.^21^^,^^22^ For this reason, we conducted further assessment of these methods using other cancer types from TCGA and an additional colorectal cancer dataset.^23^

Analysis of TCGA lung adenocarcinoma (LUAD, *n* = 495) and skin cutaneous melanoma (SKCM, *n* = 450) cohorts showed a strong correlation between OE-ratios, with a greater overlap of samples identified as immunoedited by OE-ratios, HBMR, and immune dN/dS compared with ES-CCF, although differences existed across different cancer types. The Spearman correlation coefficient between two OE-ratios reached 0.90 and 0.93 in LUAD and SKCM, respectively (*p* < 0.05, Figures 2D, S2G, and S2H; Figure S3A), while ES-CCF was weakly associated with HBMR in LUAD (Figure S2G). Immune dN/dS was positively correlated with ES-CCF in LUAD (Spearman correlation coefficient 0.11; *p* < 0.05), but negatively correlated in SKCM (Spearman correlation coefficient −0.12; *p* < 0.05). The number of immunoedited samples in LUAD identified by OE-ratios (53.53% [265 of 495]) was comparable to HBMR (54.34% [269 of 495]) (Figure 2E). In SKCM, the OE-ratios identified the largest proportion of immunoedited samples (88.67% [399 of 450], Figure S3B). ES-CCF still identified the lowest number of immunoedited samples across both cancer types (4.85% [24 of 495] for LUAD and 6.44% [29 of 450] for SKCM). In LUAD, 432 samples were identified as undergoing immunoediting by at least one method and the proportion of immunoedited samples uniformly identified by OE-ratios, HBMR, and immune dN/dS was 15.97% [69 of 432], while only 6.71% (29 of 432) were identified by ES-CCF. Similarly, in SKCM, 430 samples were identified as immunoedited, with 6.74% (29 of 430) of them detected by ES-CCF and 20.93% (90 of 430) concordantly identified by the other three methods (Figures 2F and S3C).

We then analyzed samples from an additional colorectal cancer cohort.^23^ After excluding samples with insufficient mutations, a total of 18 samples were included in the analysis. A strong correlation was maintained between the two OE-ratios (Spearman correlation coefficient 0.92, *p* < 0.05), and both exhibited a positive correlation with immune dN/dS (Spearman correlation coefficient 0.67 for ${OE\;ratio}_{refSample}$ and Spearman correlation coefficient 0.72 for ${OE\;ratio}_{codon}$, *p* < 0.05) (Figures S3D and S3E). Immune dN/dS could not be validly calculated for 27.78% (5 of 18) of the samples (Figure S3F). Among the 18 samples analyzed, 16 were identified as having undergone immunoediting. 18.75% (3 of 16) samples were uniformly detected as immunoedited by OE-ratios, HBMR, and immune dN/dS, while only 6.25% (1 of 16) of samples were identified as immunoedited by the ES-CCF (Figure S3G). Despite some variation in immunoediting scores, the OE-ratios, HBMR, and immune dN/dS demonstrated moderately higher overlap in identifying immunoedited samples across different datasets when compared with ES-CCF.

### Evaluation of quantitative immunoediting methods using PDX models generated in immunodeficient mice

Although there is growing focus on immunoediting analysis, a “gold standard” for its evaluation is still lacking. Patient-derived xenografts (PDXs) are cancer models in which tumor tissues from patients are usually implanted into immunodeficient mice.^24^ Owing to the absence of immune cells (such as T cells and B cells), immunodeficient mice are generally unable to elicit an effective antitumor immune response or immune selection.^25^^,^^26^ Moreover, patient-derived immune cells are gradually lost and inactivated as the PDX develops and propagates, rendering the PDX largely unaffected by the host’s immunosurveillance.^27^^,^^28^^,^^29^ This allows PDXs to accumulate many mutations that are subject to weak immune selection, making it a suitable control for assessing whether they undergo less immunoediting than their corresponding original tumors. So we assessed these methods by comparing the immunoediting scores of 10 pairs of first-generation PDX models and their corresponding original tumors.^19^

We observed significant shifts in the mutation profile between PDX and matched primary tumors, with shared mutation proportions ranging from 0.3% (P22) to 67% (P29, Figure S3H). Fifty percent of PDXs were primarily composed of mutations from the original tumor, while the remaining 50% were mainly composed of *de novo* mutations (Figure S3I), highlighting the ongoing genomic evolution in PDX models.^30^

**Figure 3:**
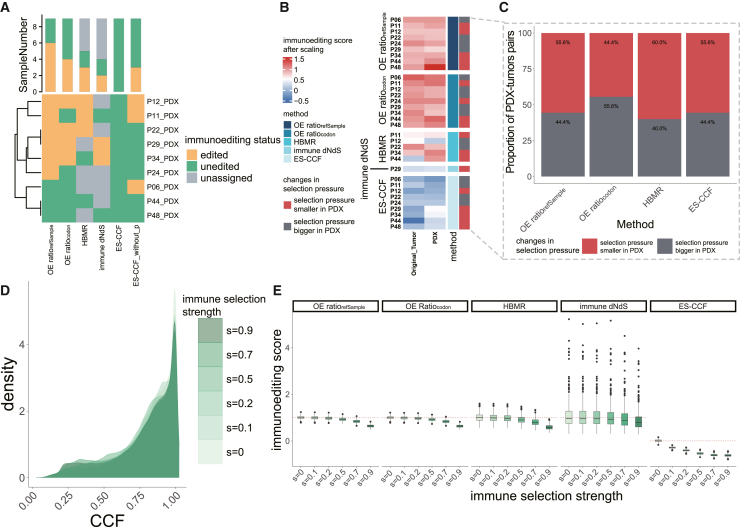
Immunoediting analysis of PDX models and simulation datasets (A) Bar chart summarizes the total number of CRC samples classified by each method as edited, unedited, or unassigned. The bottom heatmap shows the detailed categorization of each sample. ES-CCF_without_p identified samples as edited when their ES-CCF was less than 0. (B) Heatmap shows the immunoediting scores calculated by different methods across PDX models and corresponding tumors, each row represents a patient. (C) Proportion of PDX models that exhibited reduced immune selection pressure compared to their original tumors. (D) CCF distribution of mutations in the simulated dataset under different immune selection. (E) Distribution of immunoediting scores obtained by different methods for simulated datasets under different immune selection, the two-sided Wilcoxon rank-sum tests are used to compare immunoediting scores across multiple groups and *p* values were corrected using the FDR method. Immunoediting scores were significantly decreased for all methods as immunoselection was increased (*p* < 0.05). See also Figure S3.

We then quantified the immunoediting of PDXs and original tumors; samples without enough available mutations or neoantigens were excluded from analysis (samples from patient P41). Similar to the analysis using TCGA data, ES-CCF identified 100% of samples as unedited when significance is considered and 66.7% when it is not, while some samples could not be effectively quantified for immunoediting using either HBMR or immune dN/dS (Figure 3A). The proportion of PDX-tumor pairs for whom these methods identified reduced immune selection in PDX compared with matched tumors ranged from 44.4% to 60% and a total of 77.78% of PDXs identified by these methods as having lower immune selection than original tumors (Figures 3B and 3C). Among all patients, only patient P29 allowed for the quantification of immunoediting in both the PDX and the original tumor using immune dN/dS, with the PDX exhibiting weaker immune selection compared with the original tumor. However, the OE-ratios failed to detect higher immunoediting scores in the PDX from P29, implying the complementarity of these methods. This complementarity among different methods was also observed in P12, P11, and P24 (Figure 3C). Taken together, it is essential to integrate multiple methods to enhance the reliability of future quantitative analyses of immunoediting.

### Simulation-based evaluation of quantitative immunoediting methods

Analyzing synthetic datasets is another effective strategy for evaluating these quantitative methods of immunoediting. Through generating simulated datasets under different strengths of immune selection, we are able to assess whether these methods effectively reflect the changes in immune selection strength. We then generated simulation datasets under different immune selections: (1) removing neoantigens, (2) lowering the CCF of mutations, and (3) a combination of both (see STAR Methods).

When only the number of neoantigens was changed, immunoediting scores derived from ES-CCF were significantly distorted, while those obtained by other methods remained decreased with the reduction of neoantigens (false discovery rate [FDR]-adjusted *p* < 0.05, two-sided Wilcoxon rank-sum tests, Figure S3J). In contrast, when only the CCF was changed, ES-CCF was the only method sensitive to changes in immune selection (FDR-adjusted *p* < 0.05, two-sided Wilcoxon rank-sum tests, Figure S3K). This may be because these methods focus on changes in the number of mutations (OE-ratios, HBMR, immune dN/dS) or the pattern of CCF distribution (ES-CCF) to measure immunoediting. We then combined the two strategies to generate simulated datasets in different immune selections. With the gradual increase in immune selection, the number of neoantigens and non-synonymous mutations in the simulated dataset decreases, while the number of synonymous mutations remains constant (FDR-adjusted *p* < 0.05, two-sided Wilcoxon rank-sum tests), and the overall CCF declines (Figures S4A and 3D). The immunoediting analysis of the simulated datasets revealed that the immunoediting scores for all methods declined as immune selection increased, especially ES-CCF (FDR-adjusted *p* < 0.05, two-sided Wilcoxon rank-sum tests, Figure 3E), which could be attributed to our simulation approach removing neoantigens by reducing the CCF. Overall, these methods are capable of reflecting changes in immune selection pressures.

### The influence of neoantigen identification on the quantification of immunoediting

Immunogenic neoantigens play a pivotal role in cancer immunoediting, thus making the criteria for neoantigen identification crucial for quantifying immunoediting. Current screening criteria for candidate neoantigens primarily include peptide length, binding affinity to major histocompatibility complex (MHC) molecules, and gene expression levels.^31^^,^^32^^,^^33^^,^^34^ We applied these immunoediting methods to CRC samples from TCGA (*n* = 366) under different conditions for each criterion. The HBMR and immune dN/dS were excluded from this analysis because they measure immune selection independently of neoantigens.

**Figure 4:**
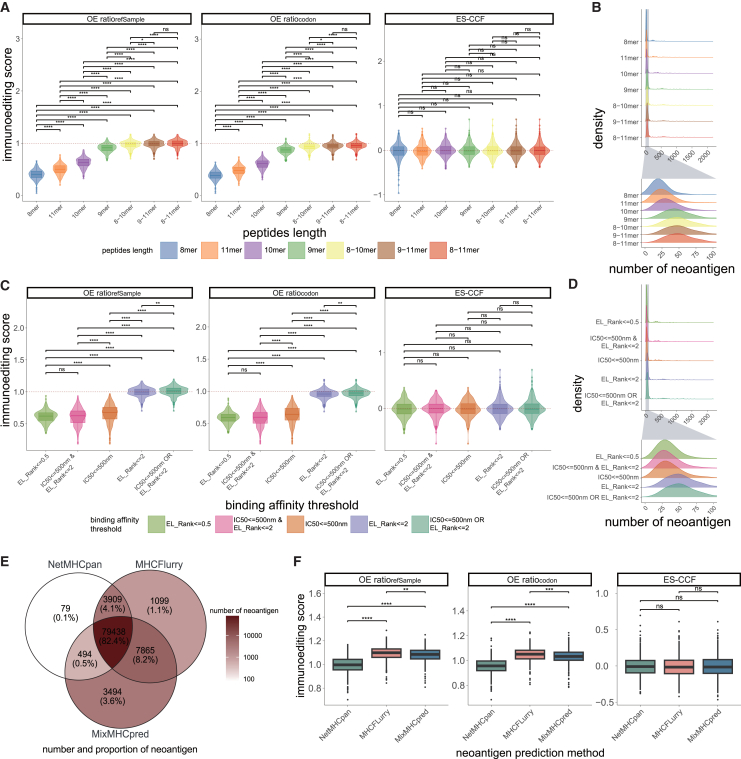
Immunoediting scores across methods under various neoantigen screening conditions (A and C) Distribution of immunoediting scores obtained by different methods for TCGA CRC samples after screening for neoantigens, distinguished by various peptide lengths (A) or binding affinities (C). FDR-adjusted *p* values from two-sided Wilcoxon rank-sum tests are indicated. (B and D) Density plot shows the number of neoantigens in CRC samples after screening for neoantigens based on different peptide lengths (B) or binding affinities (D). (E) Neoantigen sharing identified by NetMHCpan, MHCFlurry, and MixMHCpred. (F) Distribution of immunoediting scores obtained by different methods using neoantigens predicted by NetMHCpan, MHCFlurry, and MixMHCpred. FDR-adjusted *p* values from two-sided Wilcoxon rank-sum tests are indicated. Statistical significance is denoted by *p* values as ∗*p* < 0.05, ∗∗*p* < 0.01, ∗∗∗*p* < 0.001, ns = not significant. See also Figures S4.

We first analyzed the impact of peptide length on immunoediting quantification. HLA-I alleles are more likely to bind to peptides of 9–10 mer,^35^^,^^36^ whereas many studies extend the range to 8–11 mer to identify more potential neoantigens.^37^^,^^38^ We found that the immunoediting score derived from OE-ratios was significantly higher when using the peptide lengths of 8–11 mer compared with other peptide lengths (${OE\;ratio}_{refSample}$ and ${OE\;ratio}_{codon}$, FDR-adjusted *p* < 0.05, two-sided Wilcoxon rank-sum tests, Figure 4A). This trend might result from the larger number of neoantigens within the 8–11 mer peptide length range^39^ (Figure 4B). Binding affinity (half maximal inhibitory concentration [IC50]) is the strength of the binding interaction between peptide and MHC, the smaller the IC50 value, the greater the binding affinity of the peptide and MHC. In NetMHCpan, weak neoantigens are often identified by a binding affinity (IC50) of less than 500 nM or an eluted ligand (EL) rank under 2%, while strong binders are typically identified using a rank below 0.5%,^40^ which are more readily recognized by T cells than the “weak” neoantigens.^41^ Subsequently, we assessed the effect of varying affinity thresholds for neoantigen prediction on the immunoediting score. The significantly lower scores in OE-ratios and fewer neoantigens as the affinity thresholds became stricter (FDR-adjusted *p* < 0.05, two-sided Wilcoxon rank-sum tests, Figures 4C and 4D). This observation also extended to other epithelial cancers (Figures S4B and S4C). In addition to peptide length and binding affinity, RNA expression is also essential for neoantigen prediction, as expressed neoantigens are more likely to produce abnormal proteins that induce an immune response.^42^ We further evaluated the impact of neoantigens not filtered by expression compared with expressed neoantigens (median TPM ≥1 in the samples) on immunoediting quantification. A significant difference in the OE-ratios was observed between the two conditions (*p* < 0.05, two-sided Wilcoxon rank-sum tests, Figure S4D). These results indicate that neoantigen expression is a relevant consideration in the quantitative analysis of immunoediting. However, the ES-CCF method displayed minimal sensitivity to changes in peptide length, binding affinity, or gene expression levels.

Previous studies have demonstrated that various HLA-binding prediction methods display differing performance and sensitivity in neoantigen identification,^43^^,^^44^ potentially influencing the quantification of immunoediting. We further applied MHCFlurry and MixMHCpred to identify neoantigens (see STAR Methods) and conducted a comparative analysis using CRC samples. NetMHCpan, MHCFlurry, and MixMHCpred identified 83,920, 92,311, and 91,291 neoantigens, respectively, with an overlap of 82.4% (Figure 4E). The OE-ratios for neoantigens predicted by MHCFlurry were significantly higher than those for neoantigens predicted by NetMHCpan. Significant differences in the OE-ratios were also observed when using MixMHCpred to identify neoantigens, while ES-CCF remained unaffected (FDR-adjusted *p* < 0.5, two-sided Wilcoxon rank-sum tests, Figure 4F).

This analysis suggests that neoantigen recognition, as a fundamental component of immunoediting quantification, will influence the OE-ratios. Stringent neoantigen recognition criteria (e.g., reduced peptide length ranges, stricter binding affinity thresholds, or the expression requirements) lead to lower OE-ratios, while ES-CCF remains unaffected. Furthermore, the identification of high-quality neoantigens will facilitate more accurate quantitative immunoediting.

### Enhanced stability of immunoediting scores with increased mutation number

Given the variation in the number of mutations between tumor samples, it is important to explore whether the mutation number could affect the performance of these methods in quantifying immunoediting. Using CRC samples from TCGA (*n* = 366), we further evaluated the relationship between the immunoediting scores obtained by each method and the number of mutations or mutation burden (TMB).

**Figure 5:**
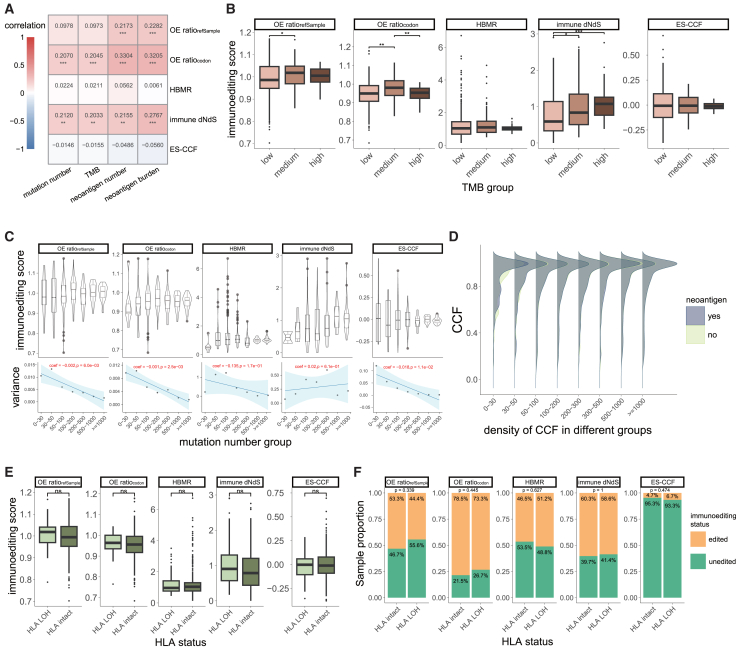
Impact of mutation number and HLA LOH on computational methods of immunoediting (A) Spearman correlation coefficients between immunoediting scores with mutation number, TMB, neoantigen number, and neoantigen burden. Correlation coefficients and significance are shown in each cell (hypermutated samples were excluded from the correlation analysis). (B) Distribution of immunoediting scores across low-, medium-, and high-TMB groups. FDR-adjusted *p* values from two-sided Wilcoxon rank-sum tests are indicated. (C) Top: Distribution of immunoediting scores obtained from different methods in each group of samples after grouping the samples according to the number of mutations. Bottom: Variance change in immunoediting scores among samples from different groups. The lines follow a fitted linear regression. Linear regression coefficients and *p* values are shown in red text. (D) Density plot shows the CCF of neoantigen and non-antigenic mutations in CRC samples. (E) Distribution of immunoediting scores obtained from different methods between samples with and without HLA LOH. *p* values from two-sided Wilcoxon rank-sum tests are indicated. (F) The proportion of samples identified as immunoedited by different methods in TCGA CRC samples with or without HLA LOH. *p* values from Fisher’s exact tests are indicated. Statistical significance is denoted by *p* values as ∗*p* < 0.05, ∗∗*p* < 0.01, ∗∗∗*p* < 0.001, ns = not significant. See also Figure S5.

We found slight but significant correlations between ${OE\;ratio}_{codon}$ and immune dN/dS with mutation number or TMB (Spearman correlation coefficient 0.20 [${OE\;ratio}_{codon}$ with mutation number], 0.21 [${OE\;ratio}_{codon}$ with TMB], 0.20 [immune dN/dS with mutation number] and 0.20 [immune dN/dS with TMB], respectively, *p* < 0.01), and the neoantigen count and neoantigen burden (the number of neoantigens per megabase) showed strong correlations with them as well (Figure 5A). We then categorized the samples into TMB-high (TMB≥20), TMB-medium (5≤TMB <20), and TMB-low (TMB<5) groups. Immunoediting scores for most methods did not show significant differences among the three groups, while ${OE\;ratio}_{codon}$ and immune dN/dS exhibited significantly higher scores in the TMB-medium and TMB-high groups, respectively (FDR-adjusted *p* < 0.01, two-sided Wilcoxon rank-sum tests) (Figure 5B). Furthermore, although there was no significant difference (FDR-adjusted *p* > 0.05, two-sided Wilcoxon rank-sum tests) when we subdivided the samples based on their mutation count, the immunoediting scores derived from OE-ratios and ES-CCF became more stable and exhibited progressively reduced variance as the number of mutations increased (linear regression coefficient <0 and *p* < 0.05, Figure 5C). The CCF distributions of antigenic mutations and non-antigenic mutations and the non-synonymous to synonymous mutation ratios in binding and non-binding regions formed the basis for ES-CCF and HBMR, respectively. Notably, these patterns and ratios showed close similarities across mutation groups, except when the mutation count in a sample exceeded 500 (*p* < 0.05, two-sided Wilcoxon rank-sum tests, Figure 5D; Figure S5A); this may explain why the immune selection pressure measured by HBMR and ES-CCF is unaffected by the number of mutations.

Additionally, analysis of hypermutated samples revealed that only the ${OE\;ratio}_{codon}$ method identified a significantly higher proportion of immunoedited samples than other methods, despite the high sensitivity of these samples to immune responses^45^^,^^46^ (*p* < 0.05, Fisher’s exact tests, Figure S5B).

### Impact of HLA alleles on measuring immunoediting

HLA alleles play a critical role in the immune response and loss of heterozygosity (LOH) of HLA-I disrupts the neoantigen presentation,^47^ potentially leading to alterations in immune selection pressure on tumors. Using TCGA CRC samples, we investigated whether samples with HLA LOH displayed elevated immunoediting scores. Although no significant differences were found, the immunoediting scores obtained by OE-ratios, immune dN/dS, and ES-CCF were slightly elevated in samples with HLA LOH (*p* > 0.05, two-sided Wilcoxon rank-sum tests, Figure 5E), and immunoediting events identified by OE-ratios and immune dN/dS were more frequent in samples without HLA LOH (*p* > 0.05, Fisher’s exact tests, Figure 5F).

**Figure 6:**
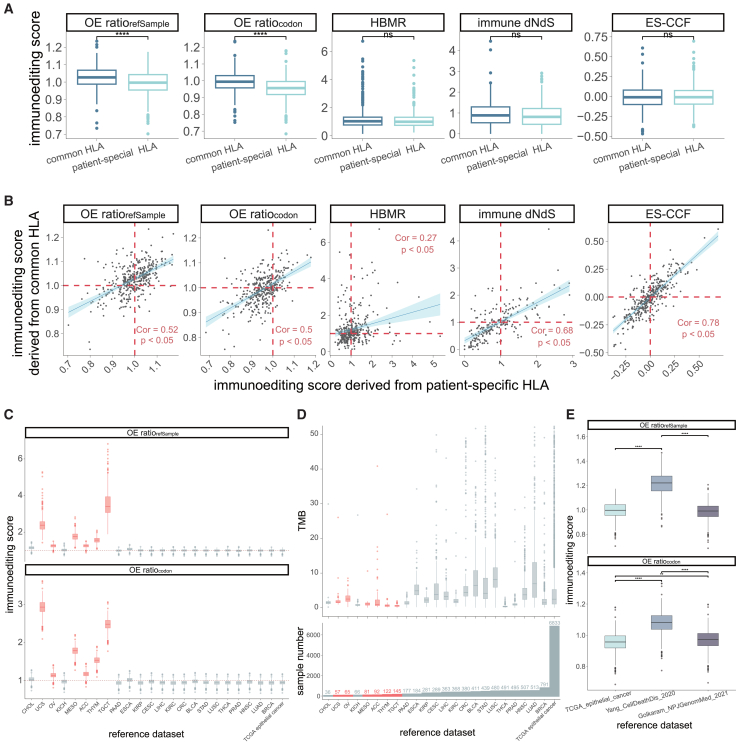
Comparison of immunoediting scores obtained with different HLA alleles selection strategies and different reference datasets (A) Distribution of immunoediting scores obtained from different methods using both common and sample-specific HLA alleles. (B) Scatterplots compare the immunoediting scores generated by common and sample-specific HLA alleles, the red dashed line represents a value of ${OE\;ratio}_{refSample}$ = 1, ${OE\;ratio}_{codon}$ = 1, HBMR = 1, or ES-CCF = 0. Spearman correlation coefficients and significance are shown. The lines follow a fitted linear regression. (C) Distribution of ${OE\;ratio}_{refSample}$ and ${OE\;ratio}_{codon}$ for TCGA CRC samples when using different reference datasets, the red dashed line represents a value of ${OE\;ratio}_{refSample}$ = 1 or ${OE\;ratio}_{codon}$ = 1. FDR-adjusted *p* values from two-sided Wilcoxon rank-sum tests among groups are demonstrated in Table S2. (D) Top: Distribution of TMB for different reference datasets from TCGA; bottom: the total number of samples for different reference datasets from TCGA. (E) Distribution of immunoediting scores for TCGA colorectal cancer samples obtained using OE-ratios with different reference datasets from other studies. Statistical significance is denoted by *p* values as ∗*p* < 0.05, ∗∗*p* < 0.01, ∗∗∗*p* < 0.001. See also Figure S6.

The selection of HLA alleles can affect the quantification of immunoediting through influencing neoantigen recognition.^48^ Previous analysis of immunoediting proposed two strategies for selecting HLA alleles, namely sample-specific HLA alleles and the most common HLA alleles.^9^^,^^15^ To evaluate the impact of the two strategies on quantification results, we annotated HLA-binding and non-binding regions for each TCGA CRC sample using sample-specific and the most common HLA alleles (HLA-A01:01, HLA-A02:01, HLA-B07:02, HLA-B08:01, HLA-C07:01, and HLA-C07:02), respectively. There were no statistical differences in HBMR between the two HLA allele selection strategies (*p* > 0.05, two-sided Wilcoxon tests, Figure 6A), and a significant correlation was observed between them (Spearman correlation coefficient 0.27, *p* < 0.05, Figure 6B), likely due to the high consistency of genomic loci identified as HLA-binding and non-binding when using sample-specific and common HLA alleles (range 65.91%–94.90%, Figure S6A). Similarly, another genomic region-based method, immune dN/dS, revealed no significant differences in immunoediting scores and maintained a high correlation under the two HLA selection strategies (Figures 6A and 6B). We also predicted neoantigens using common HLA alleles and assessed the OE-ratios and ES-CCF. Significant shifts were observed between sample-specific and common HLA alleles in OE-ratios (*p* < 0.05, two-sided Wilcoxon rank-sum tests, Figure 6A), although these metrics exhibited a moderate correlation in the two strategies (Spearman correlation coefficient 0.52 for ${OE\;ratio}_{refSample}$, 0.50 for ${OE\;ratio}_{codon}$, respectively, *p* < 0.05, Figure 6B). In contrast, ES-CCF showed a high degree of concordance (*p* > 0.05, two-sided Wilcoxon rank-sum tests; Spearman correlation coefficient 0.78, *p* < 0.05, Figures 6A and 6B). These results indicate that the influence of HLA LOH and HLA alleles cannot be neglected in the quantification of immunoediting, especially for the ${OE\;ratio}_{refSample}$ and ${OE\;ratio}_{codon}$ methods.

### Measurement of immunoediting by OE-ratios relies on population characteristics of the reference dataset

The OE-ratios can be categorized into two approaches, ${OE\;ratio}_{refSample}$ and ${OE\;ratio}_{codon}$, based on how the expected non-synonymous mutations are calculated. They use the reference dataset as a control that is not subject to immune selection pressure; however, it remains unclear whether different reference datasets affect the quantification of these methods. To evaluate this, we expanded our analysis by utilizing individual epithelial cancer types as separate reference datasets for TCGA CRC samples (*n* = 366). Compared with TCGA epithelial cancers as a reference dataset, immunoediting scores were significantly overestimated when employing reference datasets derived from uterine carcinosarcoma (UCS), OV, mesothelioma (MESO), ACC, TGCT, and THYM (Figure 6C). These cancers exhibited lower mutation burdens and smaller sample sizes relative to other references (Figure 6D). To further investigate the impact of sample size on quantification, we randomly selected varying numbers of samples from TCGA epithelial cancer dataset. There is often a significant difference in the ${OE\;ratio}_{codon}$ derived from reference datasets with large discrepancies in sample size (FDR-adjusted *p* < 0.05, two-sided Wilcoxon rank-sum tests, Figure S6B), but this difference becomes negligible when the sample size of the reference datasets exceeds 500, which indicates that selecting a reference dataset with a sufficient sample size is crucial for the OE-ratios.

Beyond TCGA data, we assessed the influence of using other external reference datasets on the OE-ratios, including colorectal cancer samples from Yang et al.^23^ (*n* = 20) and Golkaram et al.^49^ (*n* = 113). The immunoediting scores derived from the dataset of Yang et al. as a reference exhibited significant changes compared with those obtained using TCGA epithelial cancers or datasets from Golkaram et al. as references (FDR-adjusted *p* < 0.0001, two-sided Wilcoxon rank-sum tests, Figure 6E). Only ${OE\;ratio}_{codon}$ showed significant differences between TCGA epithelial cancers and Golkaram et al.’s datasets when they were used as references (FDR-adjusted *p* < 0.01, two-sided Wilcoxon rank-sum tests, Figure 6E). Taken together, these results highlight that the discrepancy of the reference dataset can influence the OE-ratios.

### How clinical variables affect immunoediting quantification

**Figure 7:**
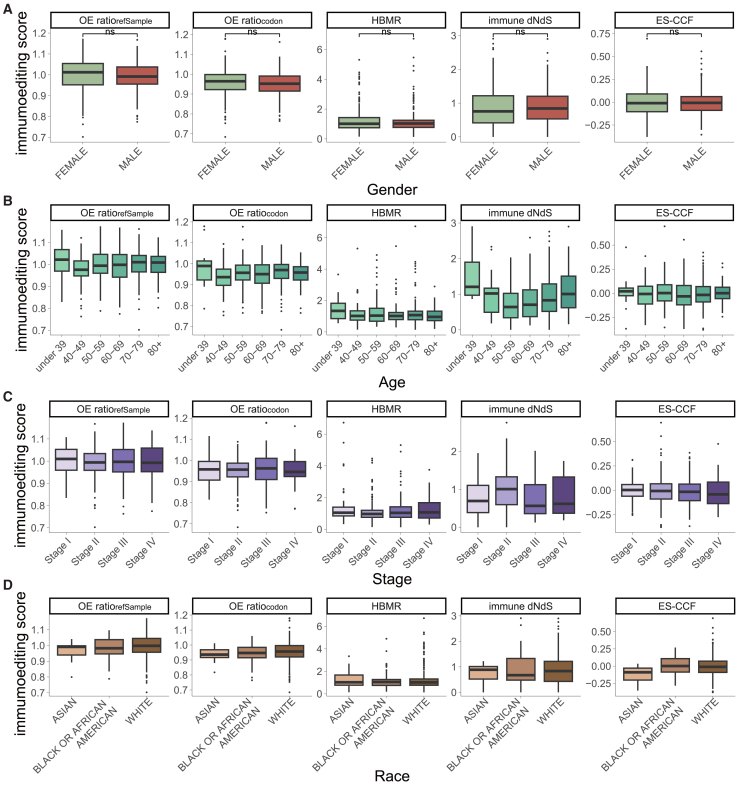
Influence of reference datasets and clinical variables on the quantification of immunoediting (A–D) Distribution of immunoediting scores by method in TCGA colorectal cancer samples, grouped by sex (A), age (B), stage (C), and race (D). Statistical significance is denoted by *p* values as ∗*p* < 0.05, ∗∗*p* < 0.01, ∗∗∗*p* < 0.001. See also Figure S7.

Previous studies have demonstrated associations between the immune environment or immune response and factors such as gender, age, disease stage, and race.^50^^,^^51^^,^^52^^,^^53^ Therefore, we subsequently investigated whether these clinical variables affect the quantification of immunoediting. First, immunoediting scores were compared between TCGA CRC samples of different genders and no significant differences were found across samples, irrespective of the methods used (*p* > 0.05, two-sided Wilcoxon rank-sum tests, Figure 7A). No significant changes in immunoediting scores were observed for these methods as age increased (FDR-adjusted *p* > 0.05, two-sided Wilcoxon rank-sum tests, Figure 7B). Further analyses of tumor stage and race also revealed no significant effects of these factors on immunoediting quantification in TCGA CRC samples using these methods (FDR-adjusted *p* > 0.05, two-sided Wilcoxon rank-sum tests, Figures 7C and 7D). Additional analyses of SKCM also did not reveal any significant alteration in immunoediting scores of each method associated with these clinical variables (Figure S7). Overall, despite observed changes in immunoediting scores, clinical variables exerted only a weak effect on immunoediting analyses in TCGA CRC and SKCM patients.

## Discussion

In this study, we systematically compared four methods for estimating cancer immunoediting and explored factors that may influence them (Figure S8). We found relatively high overlap between OE-ratios (${OE\;ratio}_{refSample}$ and ${OE\;ratio}_{codon}$), HBMR and immune dN/dS in the classification of immunoedited samples and ES-CCF tended to classify samples as unedited. Further analysis using the PDX models presented partial complementarity between these methods. In addition, OE-ratios were influenced by neoantigen selection strategies and reference datasets, and other methods showed less sensitivity to HLA alleles and the number of mutations in the samples.

The central assumption of these methods is that immune clearance results in fewer neoantigens than expected (i.e., the indicator under immune selection is lower than that unaffected by immune selection).^54^^,^^55^ However, the specific indicators employed by different methods to measure immunoediting may contribute to consistency or divergence in their quantification. For the method based on observed-to-expected neoantigen ratio, both ${OE\;ratio}_{refSample}$ and ${OE\;ratio}_{codon}$ use the neoantigen to non-synonymous mutation ratio as the indicator of immunoediting and show high concordance in immunoediting score as well.^10^^,^^14^ Although HBMR quantifies immunoediting through mutation comparisons across two genomic regions, it employs metrics similar to the neoantigen to non-synonymous mutation ratio (i.e., non-synonymous to synonymous mutation ratio), showing partial overlap with OE-ratios in scoring and detecting immunoedited samples.^15^ Another genomic region-based method called immune dN/dS, which compared dN/dS on and off the immunopeptidome, also showed a degree of consistency with both OE-ratios and HBMR. These three methods quantify immunoediting through changes in mutation counts, while ES-CCF estimates the strength of the immunoediting by comparing the CCF distribution patterns of antigenic mutations and non-antigenic mutations.^17^ Additionally, ES-CCF estimates the significance of the immunoediting score by applying random permutations to detect immunoedited samples, which further increases the difference between ES-CCF and the other methods.

Our study also revealed some factors that should be considered when applying these methods. The quantification of immunoediting by the OE-ratios is highly dependent on the external reference dataset, as shown by the impact of different datasets on the OE-ratios in our results. One obvious limitation is that the immunoediting scores obtained by the OE-ratios are inflated if the reference dataset consists of a high number of immunoedited samples.^9^ Large sample sizes across cancer types in reference datasets may help mitigate sample bias issues and the development of more diverse and representative reference datasets in future studies will enhance the utility of quantitative immunoediting. Furthermore, tailoring thresholds of the OE-ratios^11^^,^^14^^,^^56^ based on the specific characteristics of the data can potentially enhance the accuracy in identifying immunoedited tumors. The variability and biases in mutation-calling, HLA-typing, and tumor-purity estimation can also propagate errors into the quantification of immunoediting. Therefore, integrating multiple mutation-calling tools to identify consensus mutations,^57^ combining multiple HLA typing algorithms to derive consensus HLA genotypes,^58^ and incorporating digital image analysis or microscopic inspection to estimate tumor purity^59^ may help mitigate the impact of these issues on immunoediting quantification. Accurate HLA typing enhances the quantification of immunoediting by OE-ratio, immune dN/dS and ES-CCF, as these methods rely on neoantigen prediction or the annotation of the patient-specific immunopeptidome. Similarly, several algorithms exist for inferring CCF^60^^,^^61^^,^^62^ but their performance is not consistent.^63^ Given ES-CCF’s emphasis on changes in CCF distribution patterns in quantitative immunoediting, exact CCF inference will improve the effectiveness of this method.

Several other factors may influence the quantification of immunoediting, including reduced neoantigen expression,^64^^,^^65^ genetic and epigenetic alterations in antigen-presenting genes (e.g., HLA-I and B2M),^66^^,^^67^ the overexpression of classical immune checkpoint PD-L1, and changes in the tumor microenvironment (e.g., high infiltration of immune-suppressing cells).^68^^,^^69^ These factors could potentially alter immunoediting scores by hindering the antigen presentation process or inhibiting T cell activity. Besides, immune-privileged tissues, such as the eye and brain, are generally believed to exclude and silence potentially invasive lymphocytes,^70^^,^^71^ possibly resulting in weak immune selection. Recent studies indicated that HLA class II genes not only influence antitumor immune responses,^72^^,^^73^ but also that their LOH increases the risk of tumor development.^74^ Activation of CD4^+^ T cells mediated by MHC-II antigens, which is a necessary step in evoking effective antitumor immune responses,^75^ can lead to the mutations poorly bound to MHC-II being positively selected during tumorigenesis,^76^ suggesting that MHC class II-restricted CD4^+^ T cells can also edit tumor immunogenicity. Therefore, HLA class II neoantigens should be included in the quantification of immunoediting, and future research should carefully consider these factors to enhance the understanding of immune selection and improve the quality of immunoediting-related studies.

Moreover, immunoediting is a dynamic process accompanying tumor progression and can reoccur after an immunotherapeutic response.^77^^,^^78^ Longitudinal studies of tumors are needed to capture the dynamic nature of immunoediting, which will be particularly informative in revealing the complex dynamic interactions between tumors and immune components.^14^^,^^79^ Notably, TCGA repository predominantly consists of individuals of European ancestry. Studies have shown that European populations exhibit lower levels of immunosuppressive factors and immunoediting scores compared with African populations,^80^^,^^81^ which may limit the applicability of immunoediting findings in TCGA to underrepresented populations. Therefore, incorporating more diverse racial datasets,^82^^,^^83^ systematically evaluating the performance of immunoediting methods within specific racial groups, and ultimately determining whether these methods exhibit race specificity is crucial for mitigating population bias in future immunoediting analyses.

Finally, recent studies have demonstrated the prognostic value of immunoediting scores derived from ${OE\;ratio}_{codon}$ and HBMR.^14^^,^^84^ Furthermore, patients with lower ES-CCF or immune dN/dS also exhibited a significant correlation with the efficacy of immune checkpoint inhibitor (ICI) treatment.^16^^,^^17^ These results suggest that immunoediting scores could serve as a clinical biomarker, which contributes to the advancement of stratified treatment and precision medicine targeting various phases of immunoediting.^77^^,^^85^

In summary, this study presented a comparison of several current immunoediting quantification methods. The results shown in this study not only enhance our understanding but also facilitate the optimization of these methods. Integrating the strengths of these immunoediting methods, such as weighted summation^86^^,^^87^ and average rank,^88^ is essential for robust immunoediting analysis, which will offer deeper insights into tumor clonal evolution^89^ and contribute to predicting the efficacy of immunotherapy.^14^ We hope that our study will highlight some considerations in applying these methods and assist researchers in utilizing these methods more effectively.

### Limitations of the study

The present study has two limitations. First, due to the absence of an established gold standard for the quantitative analysis of immunoediting, we evaluated these methods using xenograft tumors generated in immunodeficient mice and synthetic data. Follow-up studies with more rigorous criteria and a larger number of samples are needed to further validate our findings. Second, incorporating and accounting for potential influencing factors, such as the complex immune microenvironment, HLA class II neoantigens, and the clinical characteristics of patients in future evaluations would be beneficial for advancing research on tumor immunoediting.

## Resource availability

### Lead contact

Further information and requests for resources and data should be directed to and fulfilled by the lead contact, Yun Xiao (xiaoyun@ems.hrbmu.edu.cn).

### Materials availability

This study did not generate new unique materials.

### Data and code availability

• The genetic data for patients in TCGA cohorts are accessible at https://gdc.cancer.gov/about-data/publications/mc3-2017. RNA-seq data from TCGA can be obtained from https://xenabrowser.net/datapages/. Raw data for the PDXs and their matched tumors are available upon request from the EBI with project ID PRJNA780253 (https://www.ebi.ac.uk/ena/browser/view/PRJNA780253). Raw data from Yang et al. and Golkaram et al. are available upon request from the NCBI database with project ID PRJNA588158 (https://www.ncbi.nlm.nih.gov/bioproject/PRJNA588158) and PRJNA689313 (https://www.ncbi.nlm.nih.gov/bioproject/PRJNA689313), respectively.
• This study does not report original code. Custom scripts used to automate the analyses are available upon request.
• Any additional information required to reanalyze the data reported in this paper is available from the lead contact upon request.

## Acknowledgments

This work was supported by the 10.13039/501100001809National Natural Science Foundation of China (grant number 82173321 to B.P.), 10.13039/501100001809National Natural Science Foundation of China (grant numbers U21A20196 and 31871336 to Y.X.), and HMU Marshal Initiative Funding (grant number HMUMIF-21008 to Y.X.).

## Author contributions

Y.X. and B.P. conceived and designed this research. S.H. was responsible for the data analysis and interpretation and manuscript writing. S.S. and K.L. provided critical comments and suggestions and revised the manuscript. All authors reviewed, edited, and approved the manuscript.

## Declaration of interests

The authors declare no competing interests.

## STAR★Methods

### Key resources table

**Table:** 

REAGENT or RESOURCE	SOURCE	IDENTIFIER
**Deposited data**		

TCGA mutation data	NCI’s Genomic Data Commons	https://gdc.cancer.gov/about-data/publications/mc3-2017
TCGA RNA seq data	XENA data portal	https://xenabrowser.net/datapages/
WES data of PDX	Yen et al.^19^	https://www.ebi.ac.uk/ena/browser/view/PRJNA780253
WGS data of colorectal cancer	Yang et al.^23^	https://www.ncbi.nlm.nih.gov/bioproject/PRJNA588158
WGS data of colorectal cancer	Golkaram et al.^49^	https://www.ncbi.nlm.nih.gov/bioproject/PRJNA689313

**Software and algorithms**		

DPClust (v2.2.8)	Nik-Zainal et al.^90^	https://github.com/Wedge-lab/dpclust
BWA-MEM (v0.7.17)	Li et al.^91^	https://github.com/lh3/bwa
XenofilteR (v1.6)	Roelof et al.^92^	https://github.com/NKI-GCF/XenofilteR
Mutect2 (GATK v4.4.0.0)	Aaron et al.^93^	http://www.broadinstitute.org/gatk
HaplotypeCaller (GATK v4.4.0.0)	Aaron et al.^93^	http://www.broadinstitute.org/gatk
VarScan2 (v2.4.6)	Daniel et al.^94^	https://varscan.sourceforge.net/
MuSE (v2.0)	Yu et al.^95^	https://github.com/wwylab/MuSE
Strelka (v2.9.10)	Sangtae et al.^96^	https://github.com/Illumina/strelka
SomaticSniper (v1.0.5.0)	David et al.^97^	http://gmt.genome.wustl.edu/packages/somatic-sniper/
ANNOVAR	Wang et al.^98^	https://annovar.openbioinformatics.org/en/latest/user-guide/download/
POLYSOLVER (v4)	Sachet et al.^99^	https://github.com/jason-weirather/hla-polysolver
NetMHCpan 4.1	Vanessa et al.^40^	https://services.healthtech.dtu.dk/services/NetMHCpan-4.1/
MHCFlurry (v2.1.4)	O'Donnell et al.^100^	https://github.com/openvax/mhcflurry
MixMHCpred (v3.0)	Tadros et al.^101^	https://github.com/GfellerLab/MixMHCpred
R (version 4.3.2)	The R Foundation	https://www.rproject.org/

### Method details

#### TCGA dataset

We used somatic mutations obtained from the TCGA PanCancer Atlas MC3 set for all 33 available cancer types,^18^ and the data was downloaded from NCI’s Genomic Data Commons (GDC, https://gdc.cancer.gov/about-data/publications/mc3-2017). We combined data for colon and rectal adenocarcinoma and then analyzed them as a single cancer type. Sequence information was derived from UCSC hg19 and 192 mutation contexts were defined by the mutation site as well as one base upstream and one base downstream. We kept somatic mutations labeled "PASS" in "FILTER" column in MC3 set. Mutations with at least three counts of the alternative allele and an alternative allele frequency (VAF) of at least 0.01 were included. We also required mutations to have a read depth >15 for the alternative allele. In addition, we restricted our analysis to epithelial-derived cancer and our final analysis included 5,869 cancer patients and 23 cancer types. For each mutation, cancer cell fraction (CCF) was inferred by DPClust (v2.2.8).^90^

#### TCGA RNA-seq data

TCGA bulk RNA-seq data were downloaded from the UCSC Xena browser (https://xenabrowser.net/datapages/) for all 33 cancer types. The TCGA consortium aligned RNA-seq reads to the hg19 reference genome. For each cancer type, we rescaled the corresponding coverage estimates for all genes to have a total depth of 1e6 and the expression was log2 transformed with the addition of pseudo count of 1 (log2(TPM+1)).

#### WES data processing

We collected a dataset from Peking University Health Science Center, which includes 20 colorectal cancer patients.^23^ Raw genomic data in SRA format can be obtained from the NCBI database with accession number PRJNA588158 (https://www.ncbi.nlm.nih.gov/bioproject/PRJNA588158). We also collected a colorectal cancer dataset from Golkaram et al., which is available in the NCBI database under accession number PRJNA689313 (https://www.ncbi.nlm.nih.gov/bioproject/PRJNA689313).^49^ A dataset comprising patient-derived xenografts and matched tumors was utilized to evaluate methods for measuring immunoediting. Paired tumor-PDX-normal samples from 10 oral cavity squamous cell carcinoma patients were collected. The raw genomic data can be requested and accessed on EBI with project ID PRJNA780253 (https://www.ebi.ac.uk/ena/browser/view/PRJNA780253)^19^ in the fastq file format.

Initially, whole-exome sequencing (WES) data were aligned to the UCSC human reference genome(hg38) using BWA-MEM (v0.7.17).^91^ For tumor xenograft sequence data, trimmed reads were also aligned to GENCODE mouse reference genomes(mm10). Subsequently, aligned BAM files for PDX samples were further filtered to remove reads potentially originating from mouse contamination using the R package XenofilteR (v1.6).^92^

#### Somatic mutation detection

Somatic single nucleotide variants (SNVs) and indels from tumor DNA were identified using Mutect2 (v4.4.0.0 of GATK, http://www.broadinstitute.org/gatk),^93^ HaplotypeCaller (v4.4.0.0 of GATK), VarScan2 (v2.4.6),^94^ MuSE (v2.0),^95^ Strelka (v2.9.10)^96^ and SomaticSniper (v1.0.5.0).^97^ To enhance specificity, matched normal sample filtration was employed to eliminate background germline variations and artifacts. All variations were annotated using ANNOVAR.^98^ We obtained reliable mutation calls as follows: First, variants that were called by and passed filtering by at least two callers were included in downstream analysis. Second, mutations with at least three counts of the alternative allele and alternative allele frequency (VAF) of at least 0.01 were included. Third, we also required mutations to have read depth >15 for the alternative allele. Using the filtered variants set, we employed DPClust (v2.2.8)^90^ to calculate cancer cell fraction in primary and PDX tumor groups. Samples with mutation number greater than 1,000 were classified as hypermutated.

#### HLA typing and HLA LOH prediction

HLA genotyping for MHC class-I genes (HLA-A, HLA-B, HLA-C) was performed using POLYSOLVER (v4)^99^ on the BAM file of all normal samples matched with PDX and tumor samples, following default parameters. Reads from the WES data were aligned to a genomic sequence library of all known HLA sequences (IMGT database). Subsequently, a 4-digit HLA type for each allele was inferred. Additionally, HLA data for TCGA was obtained from supplementary material provided by Zapata et al. (https://zenodo.org/records/7546705).^16^ Copy number profiles generated by the ABSOLUTE method for TCGA samples were obtained from GDC (https://gdc.cancer.gov/about-data/publications/pancanatlas). To assess loss of heterozygosity (LOH) of HLA-I, we identified segments within the chromosome 6p locus that include the HLA-A, HLA-B and HLA-C loci. Loss of heterozygosity was defined as a minor allele copy number of 0 for any HLA-I locus.

#### Neoantigen prediction

We identified potential neoantigens generated by nonsynonymous SNVs based on exome sequencing data from PDX and tumor samples. Novel 8–11mer peptides that derived from the nonsynonymous SNVs were selected. The HLA class-I epitope binding predictions were performed using the NetMHCpan 4.1.^40^ Mutations were considered neoantigens if the resulting peptides had a predicted binding affinity ≤500 nM or an eluted ligands (EL) rank percentage score ≤2% and a strong neoantigen had an EL rank percentage score ≤0.5%. We also predicted neoantigens using MHCFlurry^100^ and MixMHCpred^101^: for MHCFlurry, mutations with a peptide that had a predicted binding affinity ≤500 nM or an affinity rank ≤2% were considered as neoantigens; for MixMHCpred, mutations with a peptide that had a predicted affinity rank ≤2% were considered as neoantigens.

#### Immunoediting analysis

##### The reference sample-based method

Immunoediting analyses were performed according to previously published methods.^9^ For analyzing immunoediting across TCGA samples, we used samples from TCGA epithelial-derived cancer to generate the null model. This model was employed to determine the expected rate of mutations that were not influenced by immune selection. When analyzing PDX with matched tumors, the PDX and their specific mutations were used to construct the null model, as we assumed that these mutations were virtually unaffected by immune selection. For each mutation context s, two rates were estimated using the null model (TCGA epithelial-derived cancer samples or PDX samples): the expected number of non-synonymous mutations per synonymous mutation, ${\bar{N}}_{s}$, and the expected number of neoantigens per non-synonymous mutation, ${\bar{B}}_{s}$, which were computed as follows:

$${\bar{N}}_{s}=\left(\frac{{nonsyno}_{A\left[A>C\right]A}}{{syno}_{A\left[A>C\right]A}},\frac{{nonsyno}_{A\left[A>G\right]A}}{{syno}_{A\left[A>G\right]A}},\ldots ,\frac{{nonsyno}_{T\left[T>G\right]T}}{{syno}_{T\left[T>G\right]T}}\right)$$

$${\bar{B}}_{s}=\left(\frac{{neoantigen}_{A\left[A>C\right]A}}{non{syno}_{A\left[A>C\right]A}},\frac{{neoantigen}_{A\left[A>G\right]A}}{non{syno}_{A\left[A>G\right]A}},\ldots ,\frac{{neoantigen}_{T\left[T>G\right]T}}{non{syno}_{T\left[T>G\right]T}}\right)$$

Subsequently, for a given set of synonymous mutations in sample i ($S_{i}$), the expected number of non-synonymous mutations ($N_{pred,i}$) and the expected number of neoantigens ($B_{pred,i}$) were calculated as:

$$N_{pred,i}=\sum_{m}^{S_{i}}{\bar{N}}_{s\left(m\right)}\times S_{s\left(m\right)}$$

$$B_{pred,i}=\sum_{m}^{S_{i}}{\bar{B}}_{s\left(m\right)}\times {\bar{N}}_{s\left(m\right)}\times S_{s\left(m\right)}$$

Where s(m) represents the mutation context of the given mutation. Finally, the immunoediting score was calculated by comparing the ratio of observed to expected neoantigens per non-synonymous mutation:

OEratiorefsample=Bobs,iNobs,iBpred,iNpred,i

Where $B_{obs,i}$ is the observed number of neoantigens and $N_{obs,i}$ denotes the observed number of non-synonymous mutations for sample i. Immunoediting of the sample was deemed to have occurred when the observed rate of neoantigens per non-synonymous mutation was lower than expected (i.e., ${OE\;ratio}_{refsample}<1$).

##### The non-synonymous substitution rate-based method

This method was fundamentally similar to the reference sample-based approach, with a difference in calculating the expected number of non-synonymous mutations.^14^ For each mutation context, the probability of resulting in a synonymous codon was calculated. Given a set of coding mutations in sample i ($C_{i}$) and their corresponding mutation context, s(m), the expected number of non-synonymous mutations was calculated as follows:

$$N_{pred,i}=\sum_{m}^{C_{i}}\left(1-{\bar{P}}_{s\left(m\right)}\right)\times C_{s\left(m\right)}$$

Where ${\bar{P}}_{s\left(m\right)}$ is the probability that mutation context m would result in a synonymous codon change.

Additionally, this method also modifies the process of counting the expected neoantigen and does not take into account the mutation context. With the expected number of neoantigens per coding mutation, ${\bar{B}}_{coding}$, and set of coding mutations $C_{i}$, the expected number of neoantigens ($B_{pred,i}$) was calculated as

$$B_{pred,i}={\bar{B}}_{coding}\times C_{i}$$

Finally, the immunoediting score (${OE\;ratio}_{codon}$) was calculated by comparing the ratio of observed to expected neoantigen per non-synonymous mutation. Immunoediting of the sample was deemed to have occurred when the ${OE\;ratio}_{codon}$ was lower than 1 (indicating the observed rate of neoantigens per non-synonymous mutation was lower than expected).

To demonstrate that the ${OE\;ratio}_{refsample}$ and ${OE\;ratio}_{codon}$ were lower than expected by chance, we generated null models by randomly permuting all mutations within the TCGA CRC dataset (*n* = 1000) while preserving the original number of mutations. Permuted samples were analyzed using the same approach described above and the *p*-value for each sample was calculated as the fraction of permuted values below its real ${OE\;ratio}_{refsample}$ or ${OE\;ratio}_{codon}$.

##### The HLA-binding peptide-based method

***HLA-binding and non-binding regions:*** To annotate HLA-binding and non-binding regions, we followed the method developed by Eynden and colleagues.^15^ First, we obtained all 9-mer peptides on each coding DNA sequence position based on HGNC symbol and UCSC hg19 genome sequence information. We then got the fasta sequence for each of these 9-mer peptides and ran NetMHCpan 4.1 with the 6 most common HLA alleles (HLA-A01:01, HLA-A02:01, HLA-B07:02, HLA-B08:01, HLA-C07:01 and HLA-C07:02). Third, an aggregated binding affinity was calculated using the harmonic mean value of the lowest binding affinity of the 6 HLA alleles. We selected positions with an aggregated binding affinity <500 nM as HLA-binding regions. Consistent with the aforementioned, we also predicted HLA-binding and non-binding regions for a subset of TCGA patients using their specific HLA alleles.

We calculated HLA-binding mutation ratio (HBMR) as previously described by Van den Eynden et al.^15^ First, we estimated the total number of non-synonymous mutations located in HLA-binding regions ($N_{binding}$) and non-binding regions ($N_{non-binding}$), respectively. Similarly, the total number of synonymous mutations in both regions was also counted ($S_{binding}$ and $S_{non-binding}$). The observed HBMR was calculated as follows:

HBMRobs=NbindingSbindingNnon−bindingSnon−binding

Next, a set of simulated mutations was constructed for HBMR analysis. For each coding sequence (CDS) position, three possible types of substitution were considered and then annotated using ANNOVAR. We estimated the probability of substitution class t ($P_{t}$) using this simulated data (Note that Eynden et al. considered 96 substitution types, rather than 192 mutation contexts). The expected HBMR was calculated as follows:

HBMRexp=Nexp,bindingSexp,bindingNexp,non−bindingSexp,non−binding=∑tNbinding,t×Pt∑tSbinding,t×Pt∑tNnon−binding,t×Pt∑tSnon−binding,t×Pt

Where $N_{binding,t}$ ($S_{binding,t}$) and $N_{non-binding,t}$ ($S_{non-binding,t}$) were the total number of non-synonymous(synonymous) mutations with class t substitutions in HLA binding regions and non-binding regions, respectively.

Finally, the normalized HBMR was calculated as follows:

$$HBMR=\frac{{HBMR}_{obs}}{{HBMR}_{\exp}}$$

Immunoediting of the sample was deemed to have occurred when the observed value was lower than the expected value (i.e., HBMR <1).

We employed a permutation method to derive a null distribution to demonstrate that the observed HBMR was lower than expected by chance. Specifically, for each sample, we randomly permuted all HLA binding and nonbinding locations (*n* = 1,000) and while preserving the number of binding/nonbinding locations to generate a null model. The permuted samples were then analyzed using the same method as applied to the real samples. The *p* value for each sample was calculated as the fraction of permuted HBMR that were below its real HBMR.

##### The immunopeptidome-based method

***Construction of patient-specific immunopeptidomes:*** We annotated patient-specific immunopeptidomes with modifications of previously published methods.^16^ First, we used the EnsDb.Hsapiens.v75 R package^102^ to generate coding DNA sequence (CDS) positions for 17,902 protein coding genes (shown in Table S3). For each CDS position, all possible overlapping 9-mer peptides were obtained. Then, HLA affinities for the entire list of peptides were predicted using NetMHCpan-4.1^40^ (default parameters) with a list of HLA alleles (4-digit resolution for HLA-A, -B and -C). This list contained 436 HLA alleles from the 1000 Genomes Project cohort^103^ and TCGA cohort (https://zenodo.org/records/7546705).^16^ Peptides with a %rank <0.5 were classified as strong binders and were used to construct an immunogenic peptides database. We further utilized the Immune Epitope Database (IEDB, http://iedb.org, accessed on 08/19/2024) to filter immunogenic peptides based on the following criteria: 1) 9-mer peptides must precisely match known peptides with positive results in T cell assays and MHC ligand assays; 2) the host organism must be human; 3) the peptide must have a validated HLA-I allele that binds to it. Peptides with corresponding gene expression having a mean or median value greater than 1 TPM in the TCGA pan-cancer cohort were finally retained.

***Calculation of immune dN/dS:*** The immune dN/dS was calculated using SOPRANO (http://github.com/luisgls/SOPRANO) in ExonicOnly mode, which was developed by Zapata et al.^16^

Herein, the SOPRANO tool calculated the dN/dS,^104^ which was the ratio of nonsynonymous to synonymous mutation rate and corrected by the 192 mutation contexts:

dN/dS=NobsNsiteSobsSsite

Where $N_{obs}$ and $S_{obs}$ denote the number of observed non-synonymous and synonymous mutations, respectively. $N_{site}$ and $S_{site}$ are the number of non-synonymous and synonymous sites corrected by each mutation context.

Then, the immune dN/dS for each sample could be obtained as:

$$immune\;dN/dS=\frac{dN/dS\left(ON\right)}{dN/dS\left(OFF\right)}$$

Where dN/dS(ON) is the dN/dS derived from patient-specific immunopeptidome, dN/dS(OFF) is the dN/dS of the patient-specific non-immunopeptidome.

Immunoediting of the sample was deemed to have occurred when dN/dS(ON) was lower than dN/dS(OFF) (i.e., immune dN/dS < 1). The *p*-value of immune dN/dS was provided by SOPRAND.

##### The cancer cell fraction (CCF)-based method

The enrichment score–CCF(ES-CCF) for each sample was calculated using the R package NeoEnrichment (https://github.com/wt12318/NeoEnrichment), as provided by Wu et al.^17^

The detailed calculation process of ES-CCF for individuals was introduced below. First, this method divided the entire CCF range (0–1) into 100 equal intervals. A rank value was then assigned to each interval (i) in descending order, and each rank value was subsequently normalized as follows:

$$R_{i}=\left|\frac{L}{2}-r\right|+1$$

Where $R_{i}$ denotes the normalized rank value, r is the original rank value, and L is the total number of intervals (which equals 100).

Then, the random variable $a_{i}$ for each interval was obtained based on the number of mutations within each interval ($m_{i}$) and the interval rank ($R_{i}$). The empirical cumulative distribution of $a_{i}$ was computed by traversing $a_{1}$ to $a_{100}$ across the total number of intervals (n) using the following equations:

$$a_{i}=\frac{m_{i}\times R_{i}}{\sum m_{i}\times R_{i}}$$

$$F\left(n\right)=\sum_{i}^{n}a_{i},n=1,2,\ldots ,100$$

For antigenic mutations and non-antigenic mutations of each sample, the distributions $F_{N}\left(n\right)$ and $F_{M}\left(n\right)$ were constructed, respectively. Then Kolmogorov–Smirnov like statistics can be calculated by taking the distance (D(n)) of two distributions:

$$D\left(n\right)=F_{N}\left(n\right)-F_{M}\left(n\right)$$

The enrichment score of CCF was defined as:

ES-CCF=max(0,D(n))−|min⁡(0,D(n))|

The immunoediting will lead to down-regulation of the CCF of neoantigens, and consequently an unbalanced distribution of the CCF of neoantigens. Furthermore, immunoediting of the sample was deemed to have occurred when the ES-CCF was lower than 0 (down-regulation of the CCF of neoantigens) and *p* < 0.05, where the *p* value was calculated by a permutation method.

#### Quantification of immunoediting

##### TCGA epithelial-derived cancers

For the analysis of TCGA epithelial-derived cancers, we considered only coding single nucleotide variants (SNVs). Each sample included at least one non-synonymous mutation, synonymous mutation and neoantigen. Then, mutations without cancer cell fraction (CCF) in each sample were excluded. We calculated ${OE\;ratio}_{refsample}$, ${OE\;ratio}_{codon}$, HBMR, immune dN/dS and ES-CCF across all samples using the methods described above.

##### PDXs and matched tumors

For PDXs and their matched tumors, we assumed the unique mutations in PDXs were virtually unaffected by immune selection and used them to quantify immunoediting in PDXs. For the original tumors, we used mutations unique to them or shared with PDXs for immunoediting analysis. Similar to the TCGA epithelial-derived cancers, we quantified the ${OE\;ratio}_{refsample}$, ${OE\;ratio}_{codon}$, HBMR, immune dN/dS and ES-CCF of each sample after screening the data according to the CCF and mutation type.

##### Compared to previous research

Several studies have performed immunoediting analysis of TCGA cancers.^9^^,^^15^^,^^16^^,^^17^ We screened the TCGA data processed in this study for cancer type and neoantigen based on the analytical strategies of these previous studies. We also obtained data for both permuted and real samples provided in the supplementary material of the study by Jimenez-Sanchez and colleagues.^10^ The analysis of ES-CCF utilized the neoantigen and CCF provided by Wu et al.^17^ We calculated ${OE\;ratio}_{refsample}$ (Figure S1A, for the study by Jimenez-Sanchez et al.), HBMR (Figures S1B and S1C, for the study by Van Den Eynden et al.), ES-CCF (Figure S1F, for the study by Wu et al.) and immune dN/dS (Figures S1D and S1E, for the study by Zapata et al., only compared CRC cohort using the immunopeptidome provided in the original article, https://github.com/luisgls/SOPRANO/blob/master/immunopeptidomes/human/allhlaBinders_exprmean1.IEDBpeps.unique.bed) again for each sample and compared them with the original results.

#### Somatic mutation simulation under different immune selection

To assess whether quantitative methods of immunoediting can reflect changes in selection pressure, we simulated somatic mutations under a series of selection pressures. First, we calculated the probability of 192 mutation contexts based on the TCGA pan-cancer cohort. This probability was used to randomly generate SNVs in the coding sequences of the reference genome (version hg19). For example, the substitution A[A>G]A would be randomly assigned to a CDS position with the base sequence AAA. Then, the SNV was annotated using ANNOVAR.

For each CDS position, we obtained all possible 9-mer peptides and ran NetMHCpan 4.1 with the 6 most common HLA alleles (HLA-A01:01, HLA-A02:01, HLA-B07:02, HLA-B08:01, HLA-C07:01 and HLA-C07:02). An aggregated binding affinity was calculated using the harmonic mean value of the lowest binding affinity of the 6 HLA alleles. An SNV was considered as neoantigen if it was located at the CDS position with an aggregated binding affinity <500 nM. The binding affinity of each neoantigen was normalized to a range between 0 and 1 and denoted by a:

$$a=\frac{500-affinity}{500-1}$$

The immune response elicited by a neoantigen increases as a rises. In this way, we generated a simulated dataset containing 500 samples, each with 1,000 mutations, and used it as baseline data not subject to immune selection. Subsequently, we randomly generated CCF values for the simulated mutations based on the CCF distribution pattern from TCGA pan-cancer data.

We defined immune selection as s. When s=0, it indicates no immune selection, and when s=1, it corresponds to the strongest immune selection. The randomly generated baseline data was considered to be free from immune selection (i.e., s=0) and were used as a reference dataset in the analysis of OE-ratios. To simulate mutations under different immune selection, we modified the CCF of neoantigens in the baseline dataset according to the s and a:

$${CCF}_{s}=CCF-s\times a$$

If ${CCF}_{s}<0.2$, we considered the neoantigen as difficult to detect and removed it from the baseline data. Using this approach, simulated mutations under different immune selections were generated from the baseline data.

We also simulated the datasets with the following adjustments: 1. removing only neoantigens while keeping CCF unchanged: after removing neoantigens with ${CCF}_{s}<0.2$, the ${CCF}_{s}$ of remaining neoantigens were reconverted to original CCF; 2. lower CCF without removing neoantigens: neoantigens were retained with ${CCF}_{s}<0.2$. Finally, these simulated datasets were used to evaluate various quantitative methods of immunoediting.

### Quantification and statistical analysis

#### Statistical analysis

Statistical tests were conducted using R (version 4.3.2). In all boxplots and violin plots, *p* values for group comparisons were calculated using the two-sided Wilcoxon rank-sum tests, which do not assume a specific data distribution. The *p*-values for multiple comparisons were adjusted using the False Discovery Rate (FDR) method. All correlations and the corresponding *p* values, including those between immunoediting scores obtained by different methods, were examined with the Spearman method via the R function "cor.test". The spearman method is used because it effectively measures the monotonic relationship between two variables and is suitable when the data does not follow a normal distribution or exhibit a non-linear relationship. The two-sided Fisher’s exact test was used to compare two groups for categorical data. Detailed descriptions of all statistical analyses are also provided within figure legends.

## Supplemental information

Document S1. Figures S1–S8

Table S1. Immunoediting scores quantified by different methods across TCGA epithelial cancer samples, related to Figure 1

Table S2. FDR-adjusted *p* values comparing different groups using various TCGA cancer types as reference datasets, related to Figure 6

Table S3. A list of the 17,902 protein-coding genes used in the study, related to STAR Methods

Document S2. Article plus supplemental information
