# Supplementary material for: Comprehensive assessment of computational methods for cancer immunoediting
Source: Cell Rep Methods. 2025 Mar 24;5(3):101006. doi: 10.1016/j.crmeth.2025.101006 (PMC12049729; doi:10.1016/j.crmeth.2025.101006)
Supplement: Document S1. Figures S1–S8 [file mmc1.pdf]

Cell Reports Methods, Volume *5*

Supplemental information

Comprehensive assessment of computational methods for cancer immunoediting

Shengyuan He, Shangqin Sun, Kun Liu, Bo Pang, and Yun Xiao

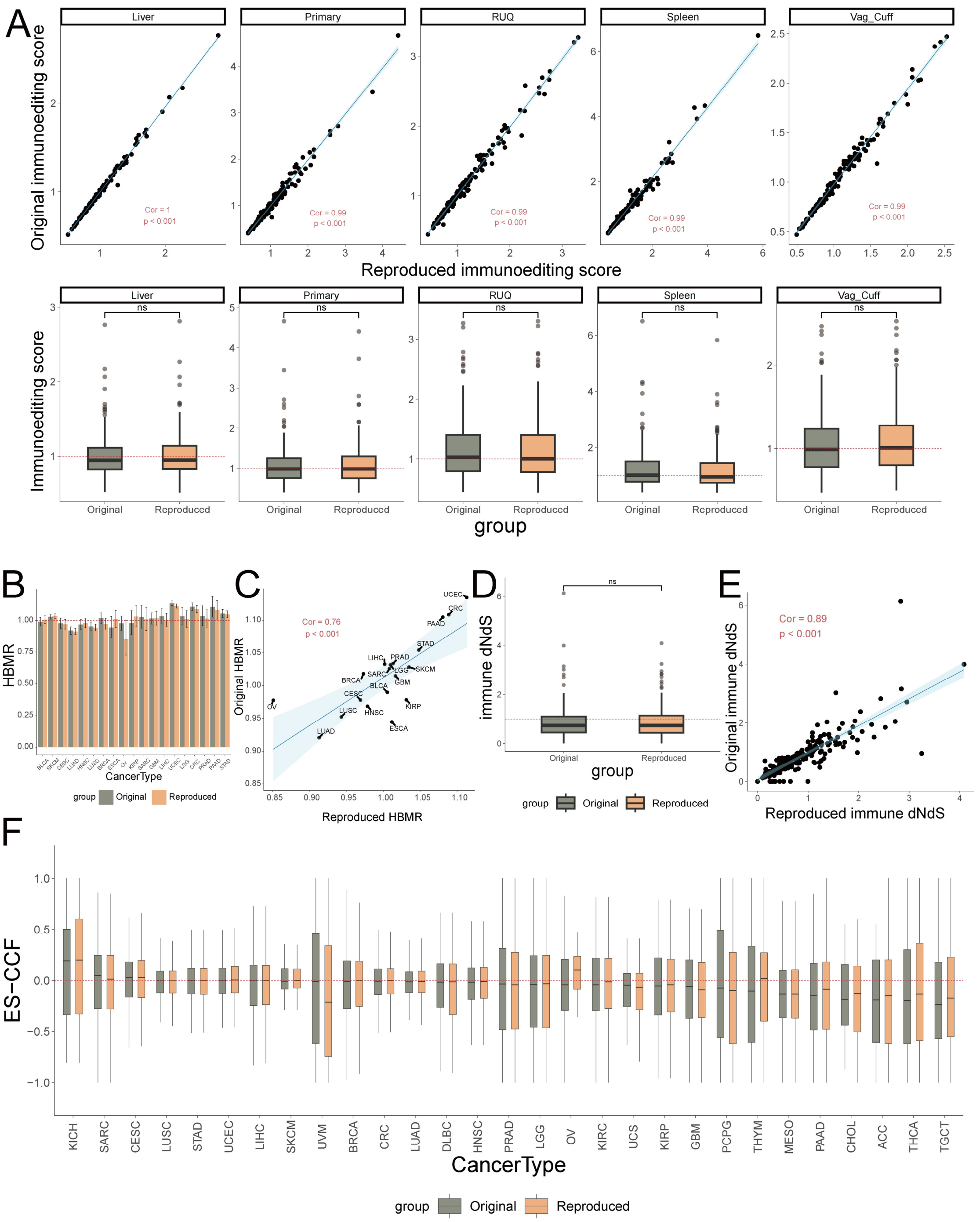

Figure S1. Reproduction and comparison of results from previous studies. Related to STAR Methods.

(**A**) Top Panel: scatter plots compare original and reproduced $OE\ ratio_{refSample}$ across different ovarian cancer samples and their corresponding simulated samples (The lines follow fitted linear regression. Spearman rank correlation and p value are shown). Bottom Panel: The distribution of original and reproduced $OE\ ratio_{refSample}$, the red dashed line represents a value of $OE\ ratio_{refSample}$ = 1 (Two-sided Wilcoxon rank-sum test p values are indicated).

(**B**) Bar chart depicts the original and reproduced HBMR across various TCGA cancer types, error bars indicate 95% confidence intervals, calculated using a two-sided Fisher's exact test, the red dashed line represents a value of HBMR = 1.

(**C**) Scatter plot shows the correlation between original and reproduced HBMR across different TCGA cancer types.

(**D**) The distribution of original and reproduced immune dN/dS in the TCGA CRC cohort, the red dashed line represents a value of immune dN/dS = 1.

(**E**) Scatter plot compares original and reproduced immune dN/dS across TCGA CRC samples. The lines follow fitted linear regression.

(**F**) The distribution of original and reproduced ES-CCF across different TCGA cancer types, the red dashed line represents a value of ES-CCF = 0. (Statistical significance is denoted by p-values as * $p < 0.05$, ** $p< 0.01$, *** $p < 0.001$, ns = not significant)

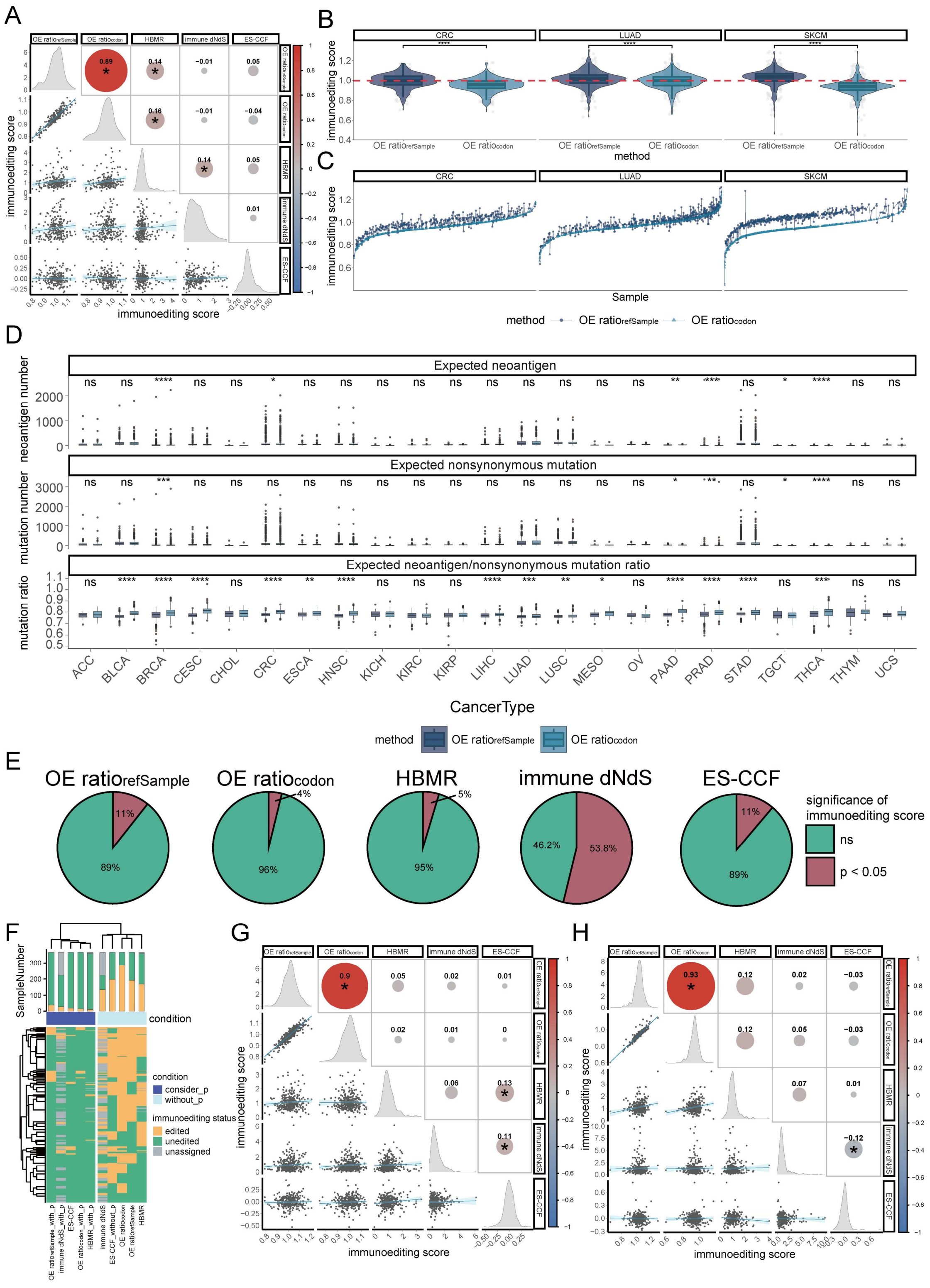

Figure S2. Detailed assessment based on colorectal cancer, lung adenocarcinoma, and melanoma from TCGA. Related to Figure 2.

(**A, G, H**) Pairwise scatterplot-matrix of immunoediting scores across TCGA CRC (A), LUAD (G) and SKCM (H) samples measured by the different methods. The diagonal presents the density curves for the distributions of each method's immunoediting scores, and the upper triangle shows the spearman correlation coefficients and p-values (The lines follow a fitted

linear regression).

(**B**) The distribution of immunoediting scores across samples measured by $OE\ ratio_{refSample}$ and $OE\ ratio_{codon}$, the red dashed line represents a value of $OE\ ratio_{refSample}$ = 1 or $OE\ ratio_{codon}$= 1 (From left to right, immunoediting score in TCGA CRC, LUAD and SKCM).

(**C**) Immunoediting scores across the TCGA CRC samples, calculated by $OE\ ratio_{refSample}$ and $OE\ ratio_{codon}$. Samples are sorted according to IE_{codon}.

(**D**) Box plot shows the expected number of neoantigens (top), non-synonymous mutations (middle) and the ratio of them across (bottom) various cancer types, comparing measurements obtained from the $OE\ ratio_{refSample}$ and $OE\ ratio_{codon}$ (Two-sided Wilcoxon rank-sum test p-values are indicated).

(**E**) The pie chart shows the proportion of CRC samples with a significantly immunoediting score measured by different methods.

(**F**) The bar chart summarizes the total number of CRC samples classified by each method as edited, unedited, or unassigned under different conditions. The bottom heatmap shows the detailed categorization of each sample under different conditions. (Statistical significance is denoted by p-values as * $p < 0.05$, ** $p< 0.01$, *** $p < 0.001$, ns = not significant)

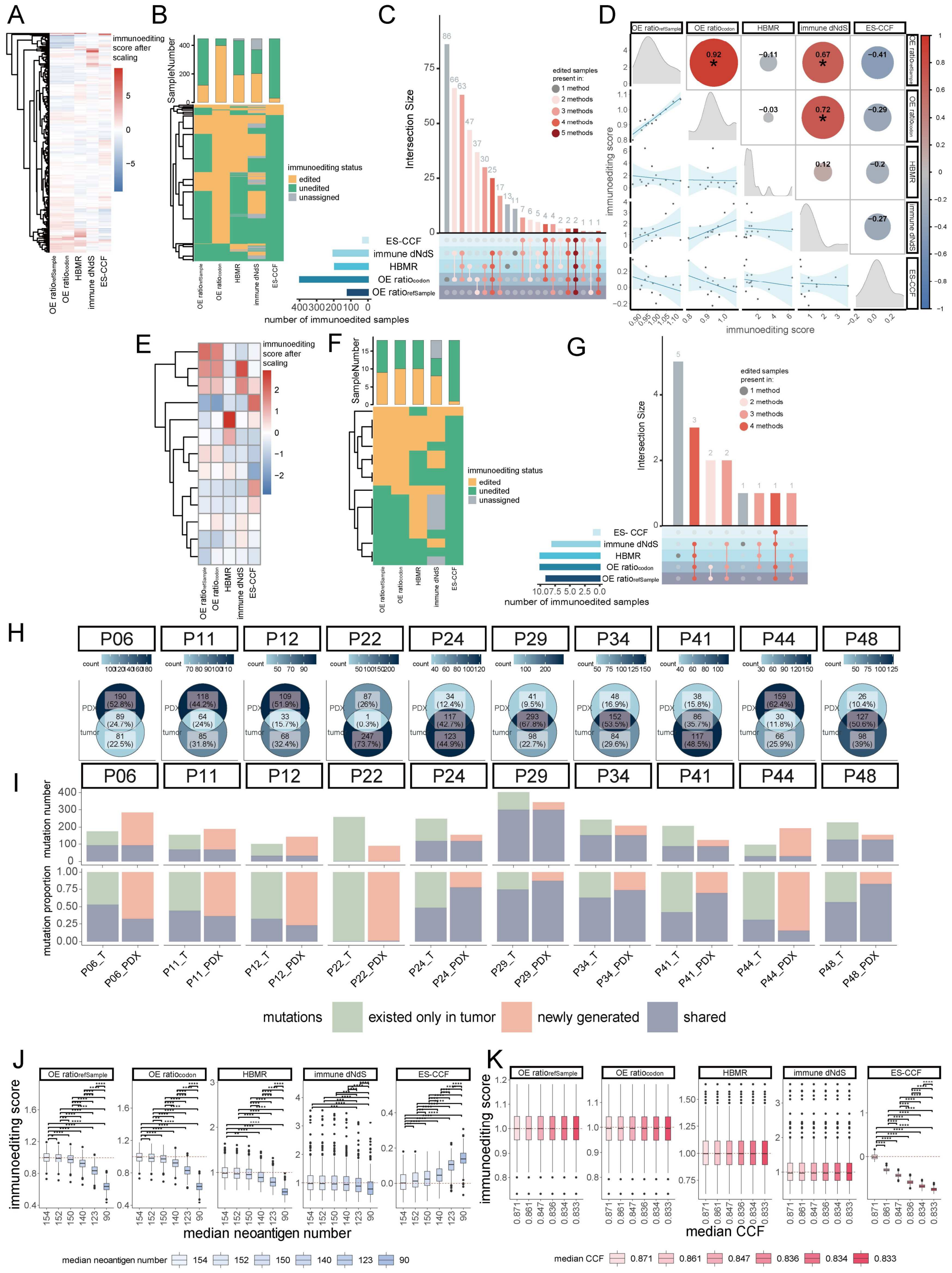

Figure S3. Evaluation based on datasets from Yang et al., PDX models, and simulated datasets. Related to Figures 2 and 3.

(**A**) Heatmap shows the similarity in immunoediting scores calculated by different methods across TCGA SKCM samples.

(**B**) Bar chart and heatmap show the total number of samples classified by each method as edited, unedited, or unassigned in SKCM.

(**C**) UpSet plot shows the intersection of immunoedited samples identified by different methods

in SKCM. The left bar plot shows the total number of immunoedited samples per method. The top bar plot shows the shared samples among methods, with color indicating the number of methods involved.

(**D**) Pairwise scatterplot-matrix of immunoediting scores across samples in dataset from Yang et al. measured by the different methods. The diagonal presents the density curves for the distributions of each method's immunoediting scores, and the upper triangle shows the spearman correlation coefficients and p-values (The lines follow a fitted linear regression).

(**E**) Heatmap shows the similarity in immunoediting scores calculated by different methods across samples in dataset from Yang et al.

(**F**) Bar chart and heatmap show the total number of samples classified by each method as edited, unedited, or unassigned in dataset from Yang et al.

(**G**) UpSet plot shows the intersection of immunoedited samples identified by different methods in dataset from Yang et al. The left bar plot shows the total number of immunoedited samples per method. The top bar plot shows the shared samples among methods, with color indicating the number of methods involved.

(**H**) Mutations shared between PDX models and their original tumors.

(**I**) Top panel: total number of mutations including those existing only in the original tumor (green), shared mutations between PDX and the matched original tumor (blue), and newly generated mutations (pink) in PDX. Bottom panel: proportion of mutations.

(**J, K**) Distribution of immunoediting scores obtained by different methods for simulated datasets under different immune selection when only neoantigens were removed (J) or only CCF was reduced (K). The horizontal axis represents the median number of neoantigens per sample (J) or the median CCF (K) in the simulated datasets. (FDR-adjusted p values are indicated, two-sided Wilcoxon rank-sum test). (Statistical significance is denoted by p-values as * $p < 0.05$, ** $p< 0.01$, *** $p < 0.001$, ns = not significant)

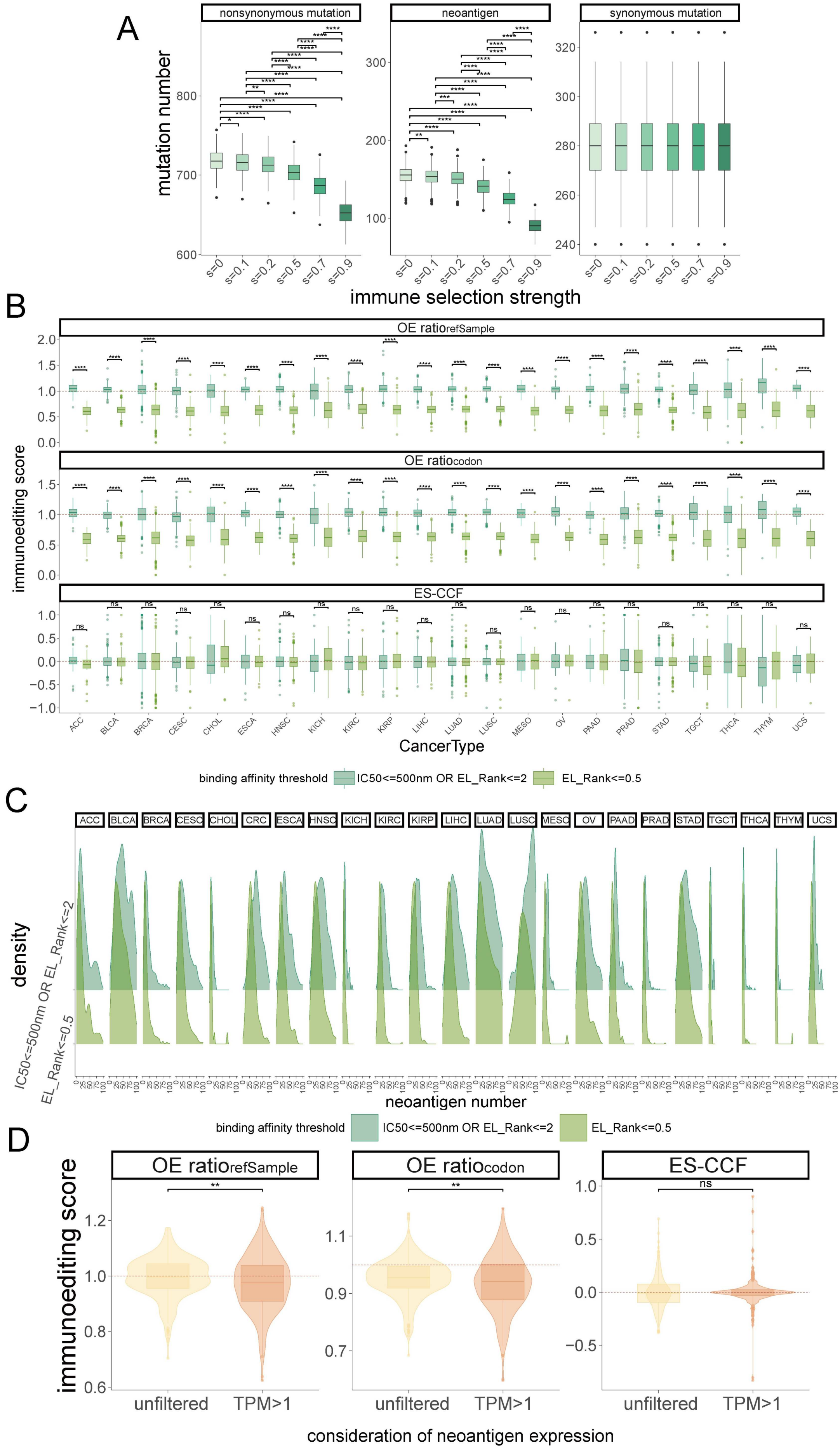

A
nonsynonymous mutation
neoantigen
synonymous mutation
mutation number
immune selection strength
s=0
s=0.1
s=0.2
s=0.5
s=0.7
s=0.9
B
OE ratiorefSample
OE ratiocodon
ES-CCF
immunoediting score
CancerType
binding affinity threshold
IC50<=500nm OR EL_Rank<=2
EL_Rank<=0.5
C
density
neoantigen number
D
consideration of neoantigen expression
unfiltered
TPM>1

Figure S4. Mutations numbers in simulated datasets and immunoediting scores obtained based on different neoantigen identification strategies. Related to Figure 3 and 4.

(**A**) Number of neoantigens, nonsynonymous mutations, and synonymous mutations in simulated data under varying immune selection. (FDR-adjusted p values are indicated, two-sided Wilcoxon rank-sum test).

(**B**) Distribution of immunoediting scores obtained from different methods applied to TCGA epithelial cancer samples, distinguished by strong or weak neoantigens. Two-sided Wilcoxon rank-sum test p values are indicated. Abbreviations: IC50, IC50 binding affinity; EL, eluted ligands.

(**C**) Density plot shows the number of neoantigens in TCGA epithelial cancer samples after screening for strong or weak neoantigens.

(**D**) Distribution of immunoediting scores obtained from different methods in TCGA CRC samples, distinguished by the expression of neoantigens (Two-sided Wilcoxon rank-sum test p values are indicated). Abbreviations: IC50, IC50 binding affinity; EL, eluted ligands. (Statistical significance is denoted by p-values as * $p < 0.05$, ** $p < 0.01$, *** $p < 0.001$, ns = not significant)

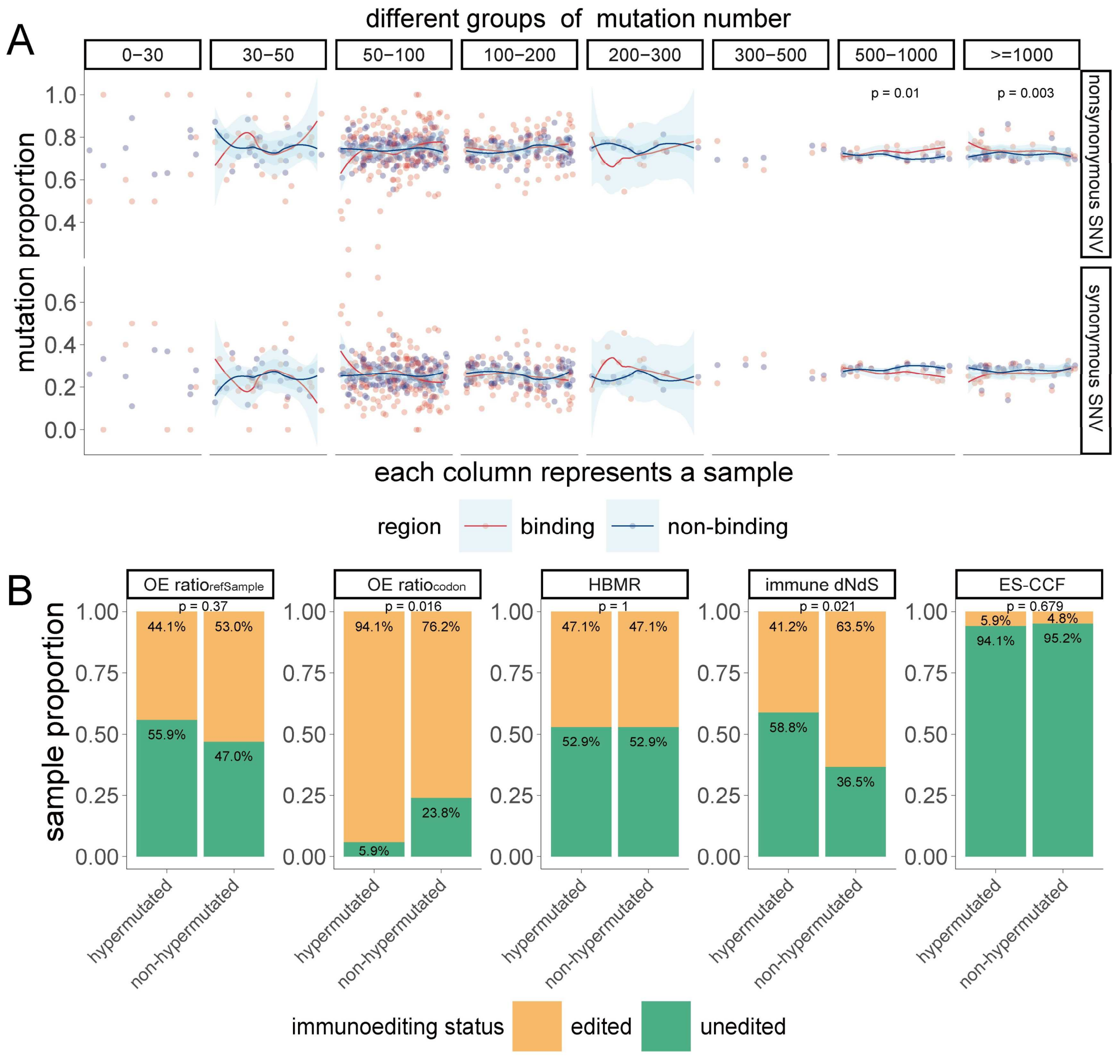

Figure S5. Impact of mutation number and TMB on the quantification of immunoediting. Related to Figures 4 and 5.

(**A**) The proportion of synonymous and non-synonymous mutations in binding versus non-binding regions across each group of samples. Fitted curves from loess regression highlight trends in subgroups with adequate sample sizes. Significant differences in the proportion of mutations within binding and non-binding regions are observed only when the mutation count exceeded 500 (Two-sided Wilcoxon rank-sum test p values are indicated).

(**B**) The proportion of samples identified as immunoedited by different methods in TCGA CRC samples with or without hypermutation (P values are calculated using a two-sided Fisher's exact test).

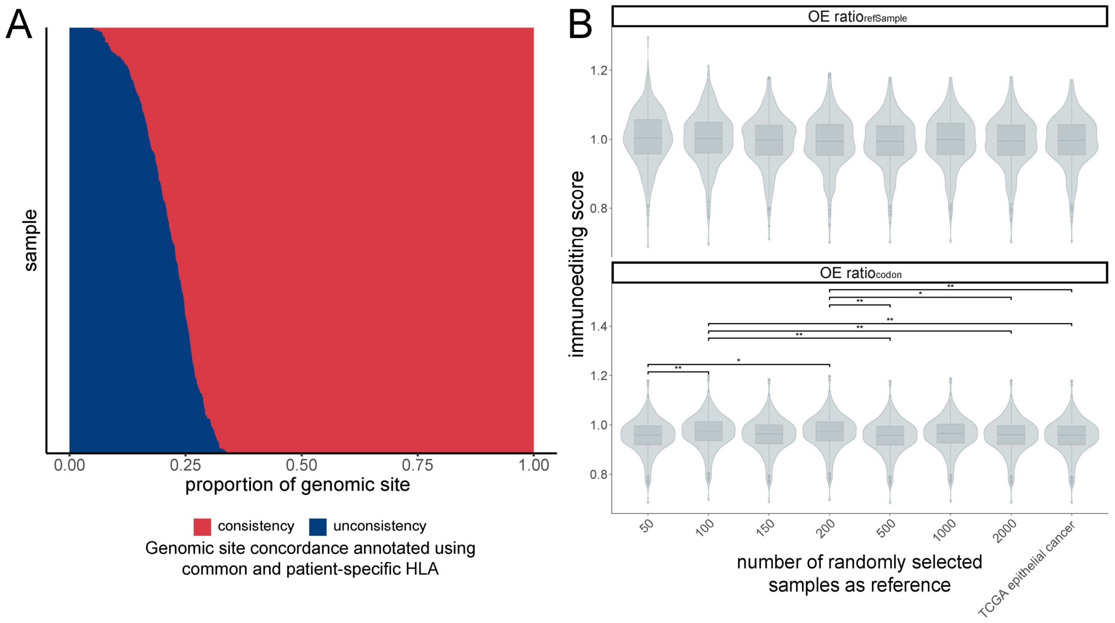

Figure S6. Impact of HLA alleles and sample size on immunoediting quantification. Related to Figure 6.

(**A**) The proportion of genomic loci with patient-specific HLA-binding and non-binding regions that are the same as those annotated using common HLA alleles.

(**B**) Distribution of OE-ratios for TCGA CRC samples when using various numbers of TCGA epithelial cancer samples as reference. (Statistical significance is denoted by p-values as * $p < 0.05$, ** $p < 0.01$, *** $p < 0.001$, ns = not significant)

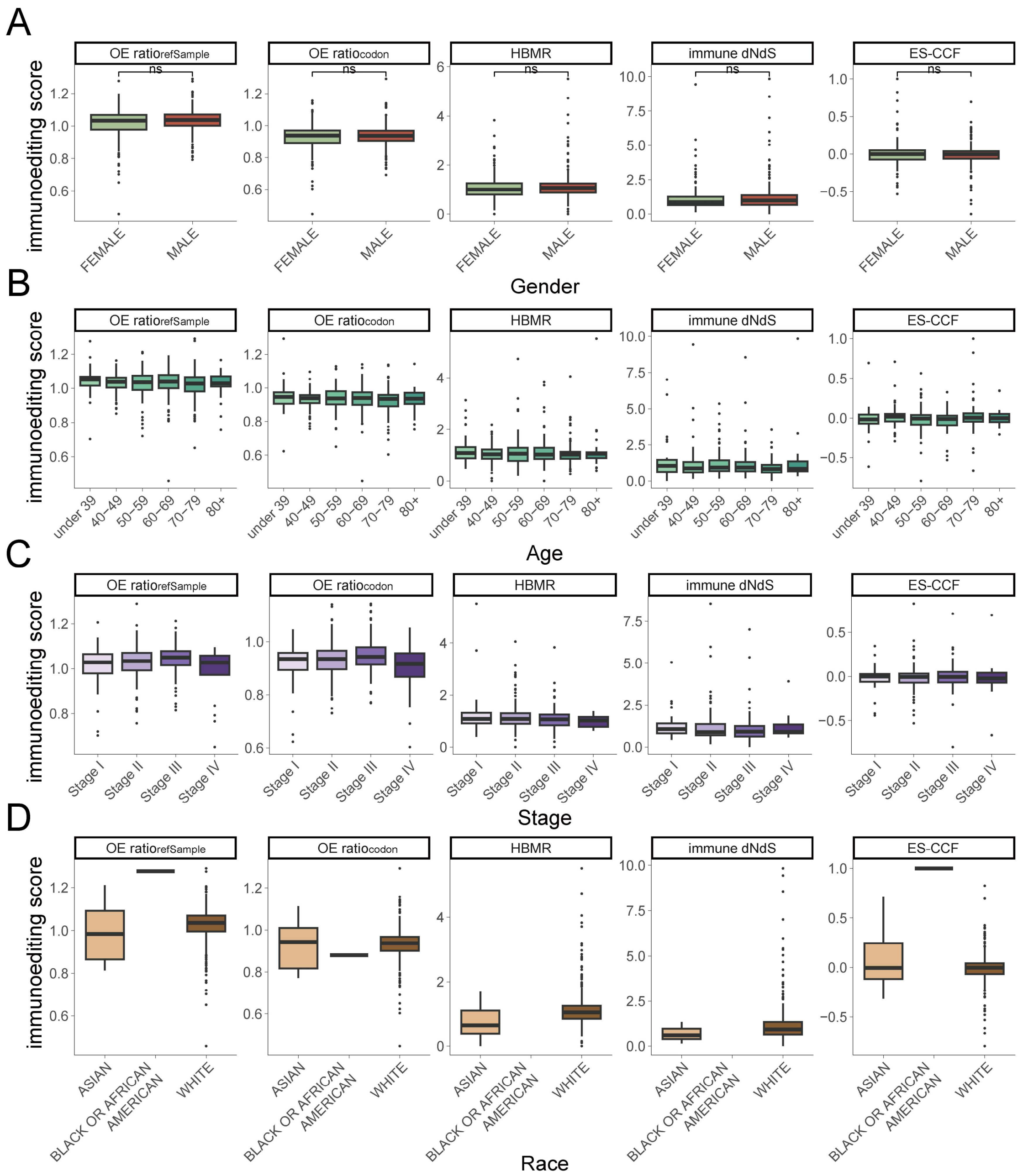

Figure S7. Impact of clinical variables on the quantitative immunoediting of TCGA melanoma samples. Related to Figure 7.

(**A-D**) Distribution of immunoediting scores by method in TCGA melanoma samples, grouped by sex (A), age (B), stage (C) and race (D). (Statistical significance is denoted by p-values as * $p < 0.05$, ** $p < 0.01$, *** $p < 0.001$, ns = not significant)

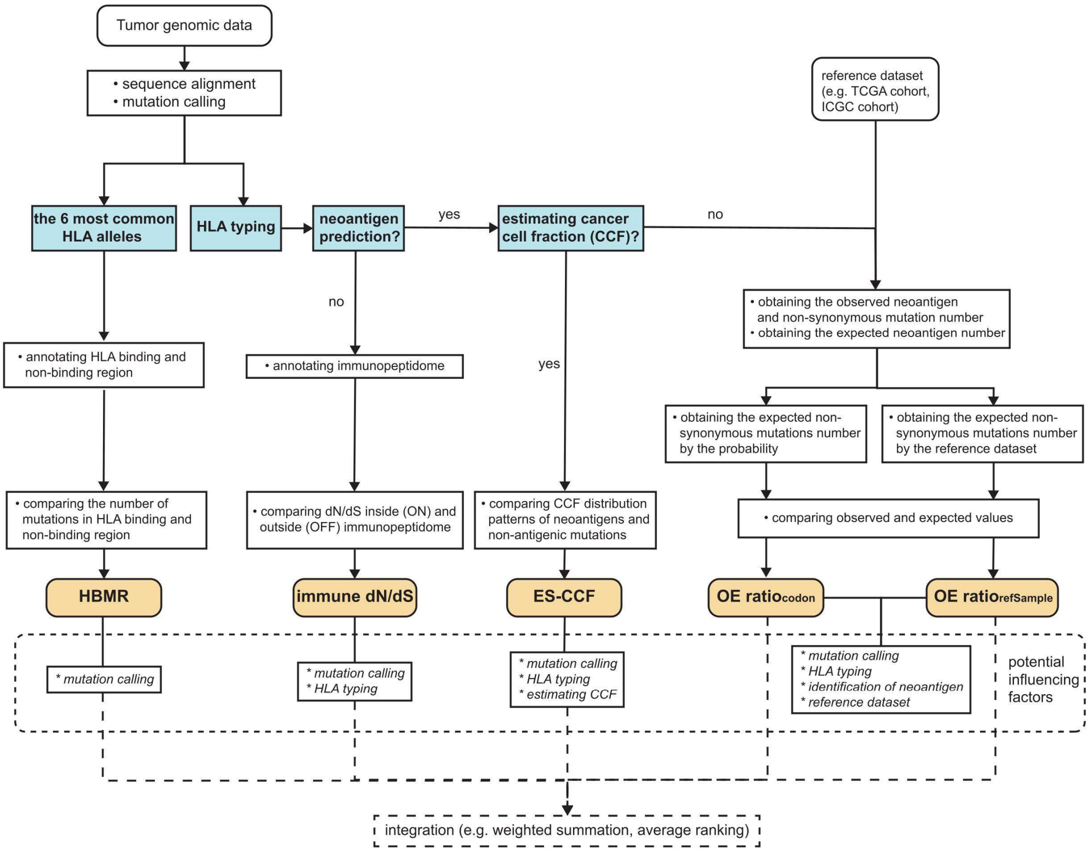

Figure S8. Flowchart for the selection and use of different immunoediting quantification methods. Related to Discussion.
